# The role of interfacial excess charge in the reversibility of proton and hydroxide solvation in electrocatalysis and bipolar membranes

**DOI:** 10.1073/pnas.2531938123

**Published:** 2026-04-16

**Authors:** Carlos Gomez Rodellar, Jody Druce, José Maria Gisbert-González, Francisco Sarabia, Beatriz Roldan Cuenya, Sebastian Z. Oener

**Affiliations:** ^a^Department of Interface Science, Fritz-Haber Institute of the Max Planck Society, Berlin 14195, Germany

**Keywords:** interfacial ion solvation, excess charge, bipolar membranes, hydrogen evolution reaction, electrochemical kinetics

## Abstract

The desolvation and recombination of protons and hydroxides in the bulk is one of the fastest reactions known to mankind. The very existence of an increased activation barrier at heterogenous interfaces reflects a key component of interfacial chemistry. Here, we use the recombination and generation of solvated $H^{+}$ and ${OH}^{-}$ at bipolar (membrane) interfaces as a test reaction to understand how interfacial excess charge impacts the electrochemical reversibility of inner-sphere reactions. We observe that excess charge, that is independent of the overpotential, primarily increases the activation energy. In contrast, when the overpotential changes the excess charge asymmetrically between the forward and reverse direction, water formation ($H^{+}+OH^{-}\to H_{2}O$) and water dissociation (WD) ($H_{2}O\to H^{+}+OH^{-}$) can switch between outer-sphere-type kinetics and distinct compensation effects between the Arrhenius pre-exponential factor and activation energy. Finally, observe that the charge transfer coefficients that quantify the overpotential dependence of the proton and hydroxide transfer through the extended hydrogen bond network are dependent on the excess charge, too, and can take values that are distinctly different from those obtained in electron-transfer reactions. The results are very important to understand how interfacial solvation and ion transfer reactions are modulated by the ubiquitous presence of excess charge across electro- and bio- and even geochemistry. Further, the results help outline the very conditions for fast electrocatalyst kinetics and describe when and how they might break down.

## Significance

The desolvation and recombination of protons and hydroxides in bulk water is one of the fastest reactions known to mankind. The very existence of an increased activation barrier at heterogenous interfaces reflects a key component of interfacial chemistry. Here, we are performing a study on the impact of interfacial excess charge on the electrochemical reversibility of the interfacial solvation kinetics and associated water dissociation (WD) and formation reactions. The results are critical to understand ion transport through the electric-field-dependent hydrogen bond network—a key component of the kinetic overpotential in inner-sphere electrochemistry.

Excess charge and electric fields are among the most central properties in heterogenous, electro- and biocatalysis. They might appear during equilibration of two phases with different electron or ion free energies or when an (electro)chemical free energy gradient (kinetic overpotential) polarizes an interface because charge transfer is impeded by an energy barrier.

In electrochemistry, the absence or minimization of excess charge is associated with ideally nonpolarizable electrodes. In this case, the reverse and forward rates are fast and establish a (reference) reduction half potential that is governed by the local thermodynamics (1), i.e. the local concentrations are described by the Nernst equation, such as for the (standard) hydrogen evolution reaction (HER) on Pt in acid. Under these narrow conditions, the reaction is driven in an overpotential range where the surface is covered with charge-compensated metal-hydrogen bonds without significant accumulation of excess charge or changes in the hydrogen coverage. Conversely, the kinetics can approximate single-step outer-sphere Marcus–Hush or Butler–Volmer theory, where increasing rates are simply driven by a decreasing activation enthalpy. In contrast, partially polarizable electrodes largely impede charge transfer and the irreversible kinetics proceed over surfaces with overpotential-dependent excess charge and intermediate coverages. However, so far, no general understanding emerged about the inner-sphere kinetics at such interfaces.

In electrocatalysis, popular understanding rests on theory that uses thermodynamic descriptors, such as the intermediate binding energy, to calculate exchange current densities and postulate volcano plots (2), with the assumption that the overpotential only reduces the activation energy, as for outer-sphere kinetics. Thus, the kinetics become “irreversible” when the activation energy at equilibrium increases and the exchange current density decreases. This reduces the range where both the forward and the reverse rate contribute to the total current.

The limitations of such simple kinetic pictures have become increasingly apparent in the recent rise of overpotential-dependent Arrhenius analysis (3–12). Our own research uncovered a ubiquitous compensation effect at low overpotentials (3–7), where an increasing Arrhenius pre-exponential factor $\left(\log_{10}A\right)$ is overcompensating an increasing apparent activation energy $\left(E_{A}\right)$. This is similar to the $\log_{10}A$-$E_{A}$ compensation reported by Conway for the HER on sp-metals (13–16), except that the latter always reported a decreasing $E_{A}$. Regardless, we followed Conway’s reasoning that compensation might arise for a dominating ion solvation step that is impacted by excess charge and the electric-field-dependent hydrogen bond network. Importantly, we correlated the activation parameters in the compensation region with operando spectroscopy performed by us and others (6, 17). However, in other work, we also showed that $\log_{10}A$-$E_{A}$-compensation can arise for multistep sequences with overpotential-dependent degrees of rate control of different rate-limiting steps and transition states (7, 18), which we discovered by ensuring excellent mass transport in the Arrhenius analysis. Thus, both electric-field effects and multisteps are unique inner-sphere aspects, but require care to isolate one from the other. This can be challenging, since multiple steps and electric fields might even arise for the seemingly simple HER (18–20).

Beyond electrocatalysis, understanding how uncompensated charge and electric fields impact an ion’s process of gaining or shedding its hydration shell is central to proton pumps and motors, ion intercalation in batteries, electrodeposition, and corrosion (21). In fact, all interfacial acid–base reactions, designated by vertical lines in Pourbaix diagrams, involve interfacial ion solvation. Therefore, understanding how excess charge impacts ion solvation is of fundamental importance to electrochemical kinetics and to form a more compressive kinetic framework that incorporates other fields, that have been believed to be separate for decades.

Bipolar membranes (BPMs) dissociate or form water into or from solvated H^+^ and OH^−^ between two ion-conducting layers ${(H}_{2}O↔H^{+}+OH^{-})$ (22). For decades, macroscopic polarization curves were fitted with overparameterized rate equations, postulating the break-down of Nernst–Planck transport, the 2nd Wien effect or other intricate physics. To overcome these approaches, we recently started to build a bridge to the solvation kinetics and general electrochemical concepts more broadly (3). Nonetheless, we still do not understand the details of how the electrochemical potential gradients of the ions inside the hydrogen bond network drive the dissociation of a neutral water molecule into solvated ions $\left(H_{2}O\to H^{+}+OH^{-}\right)$. Further, interfacial desolvation of ions and water formation $\left(H^{+}+OH^{-}\to H_{2}O\right)$ remains almost entirely unknown. Whereas, recent studies reported that water formation can be catalyzed with metal oxide species (3, 23), no detailed picture exists. In fact, it is often believed that interfacial ion transfer is just a mass transport limitation.

In the bulk, the desolvation and recombination of H^+^ and OH^−^ into H_2_O is thought to be (mass transport) limited by diffusive Grotthuss transport (24–26) across the extended hydrogen bond network, where the final recombination event was proposed by Eigen and de Maeyer to involve reorganization of the collective hydrogen bond network (27, 28). Later, Parrinello and coworkers refined this model based on extensive molecular dynamics (29, 30). In particular, it was proposed that the final recombination step occurs *via* a hydrogen bond wire spanning multiple water molecules that undergoes a collective, compressive motion (30) and that a charged, metastable state could slow down proton and hydroxide recombination (29). This understanding was refined with extensive laser spectroscopy (25, 26, 31, 32), whereas limitations of computational power remain a bottle-neck in the interpretation of complex spectroscopy signals from interfacial solvents (31). This computational limitation has slowed research in vastly different areas in physical and biochemistry, but might soon be overcome, as recent computational advances using neural networks started to capture long-range electrostatic interactions (33).

Here, we isolate interfacial proton and hydroxide generation and recombination ${(H}_{2}O↔H^{+}+OH^{-})$ at polymeric and metal–solution interfaces to understand how interfacial excess charge impacts the reversibility of the ion solvation kinetics. The results are very important to understand the differences between chemistry in the bulk and at interfaces and to provide a comprehensive picture in electrochemistry that does justice to the interfacial ion transfer kinetics.

## The Temperature Dependence of Electrochemical Kinetics

Electrochemistry provides access to the kinetics *via* the temperature-dependent current density as a function of kinetic overpotential, j(η). For an anodic current density, the Arrhenius equation with the ideal gas constant, R, writes

[1] $$j_{a}=A\left(\eta \right)\;\exp\left(\frac{-E_{A}\left(\eta \right)}{\mathrm{RT}}\right).$$

The Arrhenius pre-exponential factor, $\log_{10}\;A\left(\eta \right)$, can be obtained *via* extrapolation to infinite temperatures from the y-intercept and the apparent activation energy, $E_{A}\left(\eta \right)$, from the slope in the experimental temperature range in Arrhenius plots (log_10_ *j* vs *T*^−1^).

To quantify the overpotential dependence directly, we can introduce the apparent activation energy, ${E_{A}}_{0}$, and pre-exponential factor, $A_{0}$, at electrochemical equilibrium

[2] $$\begin{array}{c} j_{a}=A_{0}\;\exp\left(\frac{\alpha_{A}F\eta }{R}\right)\exp\left(\frac{-{E_{A}}_{0}+\alpha_{E}F\eta }{\mathrm{RT}}\right). \end{array}$$

with the component of the prefactor, $\alpha_{A}$, and apparent activation energy, $\alpha_{E}$, and define the general charge transfer coefficient

[3] $$\alpha \left(T,\eta \right)=\alpha_{E}\left(\eta \right)+\alpha_{A}\left(\eta \right)T.$$

In this notation, $\alpha_{E}\left(\eta \right)$ and $\alpha_{A}\left(\eta \right)$ remain sufficiently flexible to incorporate the multistep and apparent nature of Arrhenius analysis, while they condense to Conway’s enthalpic, $\alpha_{H}$, and entropic components, $\alpha_{S},$ for a single-step reaction, respectively (13). As we detail later, the situation is nuanced for ionic reactions, as ionic multi-steps might underlay a nominally single-step process. Regardless, for a single-step reaction, $A\left(\eta \right)$ can be linked to an overpotential-dependent activation entropy ${(\Delta S}^{\#}\left(\eta \right))$ and $E_{A}\left(\eta \right)$ on the activation enthalpy $\left({\Delta H}^{\#}\left(\eta \right)=E_{A}\left(\eta \right)-RT\right)$. Compared to Arrhenius analysis, the transfer coefficients emphasize the “spacing,” i.e. overpotential dependence of the activation parameters in the electrochemical Constable plots (kinetic maps). However, for high quality data, both approaches provide the same information, as detailed later.

The overpotential and temperature-dependent charge transfer coefficient can be obtained from the respective Tafel slope $b\left(\eta ,T\right)$ in V dec^−1^

[4] $$\alpha \left(\eta ,T\right)=\frac{\ln\left(10\right)RT}{Fb\left(\eta ,T\right)}.$$

Historically, the observation of constant (temperature-dependent) Tafel slopes over decades of currents suggested a single rate-limiting step (13, 21, 34, 35). This reasoning was extended to shorter Tafel fragments to study the number of electrons before or after a rate-determining electron transfer step (35). However, for (nominally) single-step ionic processes across the double layer, the focus on constant Tafel slopes with discrete values is not warranted (21, 35, 36). Similarly, Tafel slopes can be overpotential dependent due to overpotential-dependent rate-limiting steps (7, 18). All of these points are currently largely neglected in a literature that overemphasizes the study of linear Tafel slopes. Regardless, the need for high overpotentials to observe (potentially) constant Tafel slopes renders this approach unsuitable to interrogate kinetic limitations that arise at low overpotentials. Here, we mainly extract $\alpha \left(\eta ,T\right)$ by constrained-fitting in the reversible region and later show that overpotential-dependent Tafel slopes and transfer coefficients lead to the same information as overpotential-dependent Arrhenius analysis for high quality data. See *SI Appendix*, Supplementary Note 1 for more background on the kinetics.

We note that the dynamic microscopic environment around the fluctuating active sites remains inaccessible in this study here and requires operando spectroscopy and microscopy and advanced molecular dynamics simulations. The above kinetic framework evaluates how the ensemble properties give rise to the apparent activation parameters and steady-state currents. Further, in this whole study, the principles of microscopic reversibility and detailed balance at electrochemical equilibrium hold, i.e. there exists a reverse process that balances the forward rate. Here, we study the degree of electrochemical reversibility, which relates to the extent of the current and overpotential range, where the forward and backward reactions are substantially contributing to the total current and the local reactant and product concentrations are approximately given by the Nernst equation.

## Reversible HER-HOR Kinetics in a H_2_ Pump Cell

**Fig. 1.**
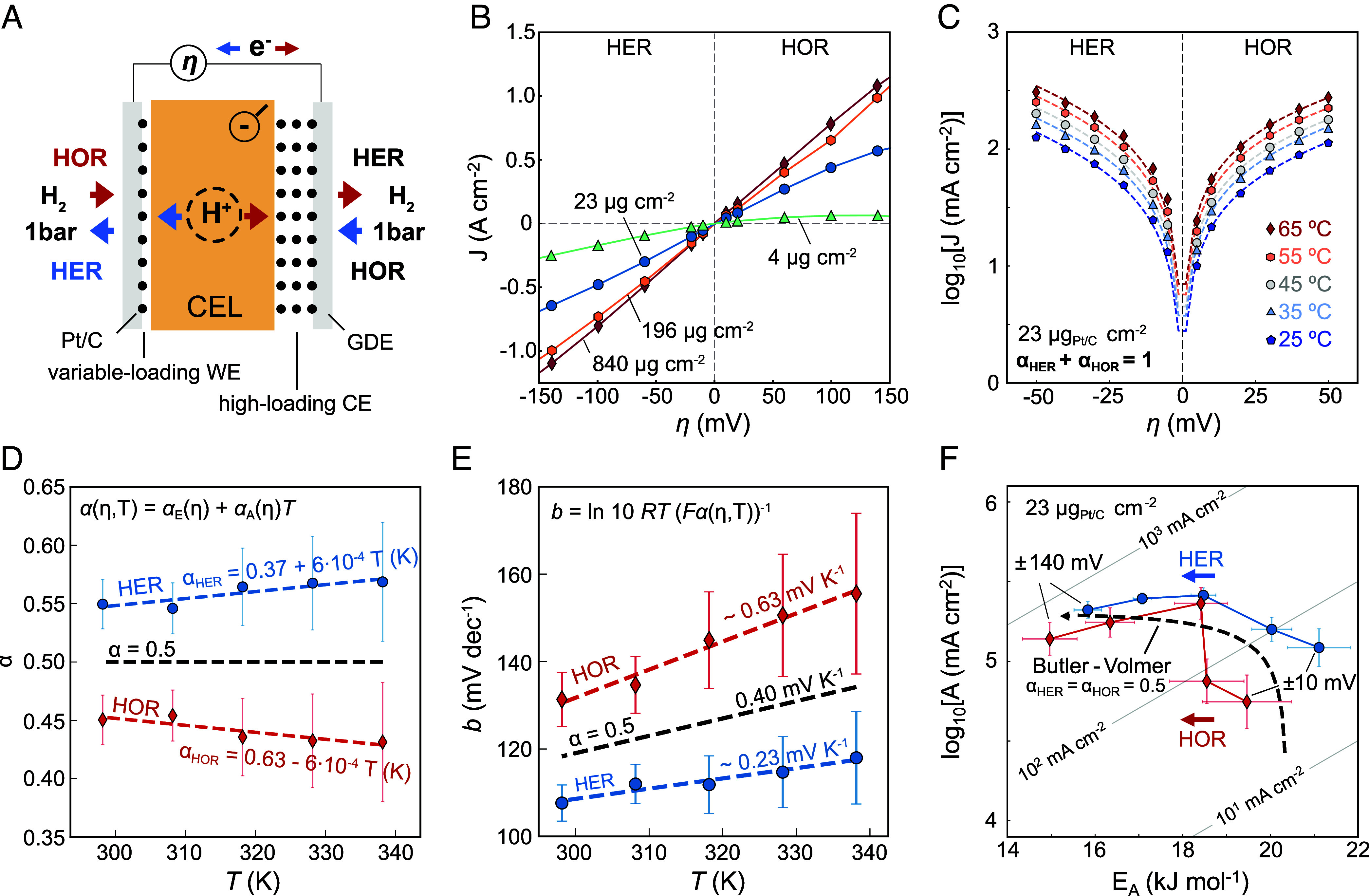
HER/HOR kinetics for reversible Pt/C electrodes in a H_2_-pump MEA. (*A*) Schematics of the H_2_-pump membrane electrode assembly (MEA), which consists of two Pt/C-loaded gas diffusion electrodes (GDEs) and a Nafion 212 (50 µm) CEL. By changing the polarity of the overpotential, the HER and HOR are studied on the low loading WE with the HOR and HER on the high loading CE, respectively. (*B*) Polarization curves for different Pt mass loadings of the WE. For 23 µg_Pt_ cm^−2^, the polarization curve is symmetric and susceptible to the loading, indicative of catalyst limited kinetics. (*C*) For 23 µg_Pt_ cm^−2^, the exchange current density,$j_{0}$, and the transfer coefficients, $\alpha_{\mathrm{HER}}$ and $\alpha_{\mathrm{HOR}}$, are obtained by fitting the Butler–Volmer rate equation to the experimental currents (±50 mV) with the constraint $\alpha_{\mathrm{HER}}+\alpha_{\mathrm{HOR}}=1$, which cannot be applied to any reaction but only to the present case as detailed in the main. Error bars are extracted from the linear regression by fitting 12 potentials. (*D*) Temperature-dependent transfer coefficients, $\alpha_{\mathrm{HER}}$ and $\alpha_{\mathrm{HOR}}$, providing $\alpha_{E}$ and $\alpha_{A}$ for each branch separately. (*E*) Tafel slope, $b=\ln10RT/(F\alpha \left(T\right))$, calculated from the temperature dependence of $\alpha_{\mathrm{HER}}$ and $\alpha_{\mathrm{HOR}}$ according to $\alpha \left(T\right)=\alpha_{E}+T\alpha_{A}$. $\alpha =\alpha_{A}=0.5$ is constant with temperature, corresponding to a Tafel slope of ~120 mV at 25 °C with a temperature dependence of ~0.40 mV K^−1^, supporting the single step assumption in the reversible regime, i.e. $\alpha_{E}=\alpha_{H}$ and $\alpha_{A}=\alpha_{S}$. (*F*) Overpotential-dependent log_10_A vs. E_A_ and calculation using a temperature-independent $\alpha =\alpha_{E}=0.5$. At low η, the suppression of the backward rate results in an apparent increase of logA. Iso-current lines are for 65 °C. Error bars are based on linear regression by fitting five currents from five temperatures.

We start with overpotential-dependent Arrhenius analysis of the HER and hydrogen oxidation reaction (HOR) on Pt/C in acid media, as a single-step test reaction approaching an ideally nonpolarizable electrode with reversible kinetics. For a simple schematic of a polarizable vs. a nonpolarizable metal–solution interface, see *SI Appendix*, Fig. S1. This extra step is needed to understand how the reversible kinetics manifest themselves in the overpotential-dependent activation parameters. For fast reactions, such as the HER in acid, previous studies have highlighted the need of providing high mass transport for accurate analysis (37–39). Thus, we use a H_2_-pump membrane electrode assembly (MEA) to measure the fast HER/HOR kinetics (Fig. 1*A*). A low loading Pt/C working electrode (WE) and gas diffusion electrode (GDE) are separated by an acidic cation exchange layer (CEL) from a high loading Pt/C counter and internal reference electrode. This follows our prior work on MEA-based Arrhenius analysis (3, 5, 7). See *Methods* for details.

Different Pt loadings in the WE were used to study the different response (Fig. 1*B*). When the same Pt loading is used in the WE as in the CE (840 µg_Pt_ cm^−2^), the linear polarization response is limited by proton transport through the CEL. In fact, using this high loading, we estimate the Nafion 212 conductivity to be 42 mS cm^−1^ at 65 °C, consistent with a typical four-point conductivity value at 60 °C and 60% relative humidity (40). With decreasing WE loading, the current decreases, transitioning to a regime limited by the HER/HOR kinetics for sufficiently low Pt loadings (23 µg_Pt_ cm^−2^ and 4 µg_Pt_ cm^−2^). For the lowest loading (4 µg_Pt_ cm^−2^), there is a substantial asymmetry in the I-V response. Lateral proton transport across the ionomer to sparse nanoparticles might be impacted by different water transport conditions between HER and HOR (41). Irrespectively, the loading of 23 µg_Pt_ cm^−2^ primarily informs on the HER/HOR kinetics. Impedance supports this selection, as shown in *SI Appendix*, Fig. S2.

The total current density, j, can be fitted to the Butler–Volmer equation when the forward and reverse rate are available, obtaining j_0_ and α directly. Fig. 1*C* shows the result for the 23 µg_Pt_ cm^−2^ loading in the range of ± 50 mV for each temperature, constraining $\alpha_{\mathrm{HER}}+\alpha_{\mathrm{HOR}}=1$, in line with previous studies (37, 38). The validity of the fitting and constraining α are detailed elsewhere (42, 43), but supported a posteriori by our results. Note that this can only be done for highly reversible reactions (21, 35). The study of reactions in their electrochemically reversible regime reduces the impact of a significant overpotential-dependent coverage variation, as well as mass-transport limitations at higher overpotentials (42, 43). Further details in *SI Appendix*, *Methods*.

Fig. 1 *D* and *E* shows the temperature-dependent charge transfer coefficient, α, and Tafel slopes, b, centered around α=0.5 and $b=120\;{\mathrm{mVdec}}^{-1}$ at 25 °C, respectively. Within experimental error, this is consistent with a reversible single-step reaction that is enthalpically driven and the assumption of a rate determining Volmer step (H^+^ + Pt ↔ Pt-H) (38). This can be seen in Fig. 1*F*, which shows the overpotential-dependent and symmetrically changing $\log_{10}\;A(\eta )$ and $E_{A}\left(\eta \right)$ that were extracted from linear Arrhenius analysis with high R^2^ linear regression values (*SI Appendix*, Fig. S3). At low overpotentials, the apparent $\log_{10}\;A(\eta )$ increases for both HER and HOR. However, no entropic or coverage effects are needed to explain this effect, which simply arises due to the suppression of the reverse rate and is fully captured by a temperature-independent $\alpha_{\mathrm{HER}}=\alpha_{\mathrm{HOR}}=0.5$ (*SI Appendix*, Fig. S4). Thus, under these single-step conditions, we can set $\alpha_{E}=\alpha_{H}$ and $\alpha_{A}=\alpha_{S}$. This exemplifies the importance of considering the reverse reaction when studying active catalysts at low overpotentials. At higher overpotentials, the apparent $E_{A}\left(\eta \right)$ is reduced, with $E_{A}^{\mathrm{HER}}\left(\eta \right)∼E_{A}^{\mathrm{HOR}}\left(\eta \right)$ and the pre-exponential factor remains approximately constant. In general, when studying highly active kinetics, even lower overpotentials should be resolved for better data quality. However, this is currently not feasible with our membrane-based setup.

The traditional Butler–Volmer equation finds its origins in early attempts to understand the (inner-sphere) HER (44), but in its traditional form with $\alpha_{H}=0.5$ and $\alpha_{S}=0$ it has no general validity in electrocatalysis (45). It always requires temperature-dependent studies to assess whether the kinetics can be approximated by outer-sphere electron transfer. Here, consistent with previous results (5, 37, 38, 46), we reason that the original Butler–Volmer equation (with $\alpha_{H}=0.5$ and $\alpha_{S}=0$) not only holds for single-step outer-sphere reactions (47), but approximately also for highly reversible single-step HER kinetics on Pt group metals with $\alpha =\alpha_{H}=0.5$ and $b=120\;{\mathrm{mVdec}}^{-1}$ at 25 °C. This is thought to be possible due to beneficial coupling of the Pt d-band with the 1 s orbital of the solvated H^+^ (48), i.e. the free energy of the solvated proton can be effectively tuned by the electron’s electrochemical potential in the metal. Conversely, the overpotential dependence is dominated by the electron transfer and the hydrogen binding energy becomes a critical descriptor—for Pt group metals (2). Similarly, the enigmatically rapid Ag-electrodeposition proceeds via Butler–Volmer kinetics (49), likely due to strong orbital interactions between the electrode and the closely approaching solvated Ag^+^ (50).

Finally, for the narrow conditions observed here, outer-sphere Marcus–Hush-type theory might provide a foundation from first principles for some aspects of the inner-sphere transfer of the electron (51–53). However, Marcus theory remains fundamentally limited not only to single-step electron transfer but also solvent reorganization terms in the inner- or outer shell of redox centers that occur before an electron tunneling event. These outer-sphere solvent reorganization terms should not be confused with ion transfer through the electric field–dependent hydrogen bond network, which is also often said to reorganize in response to the electric field.

## The Impact of Overpotential Independent Excess Charge in Bipolar Junctions

**Fig. 2.**
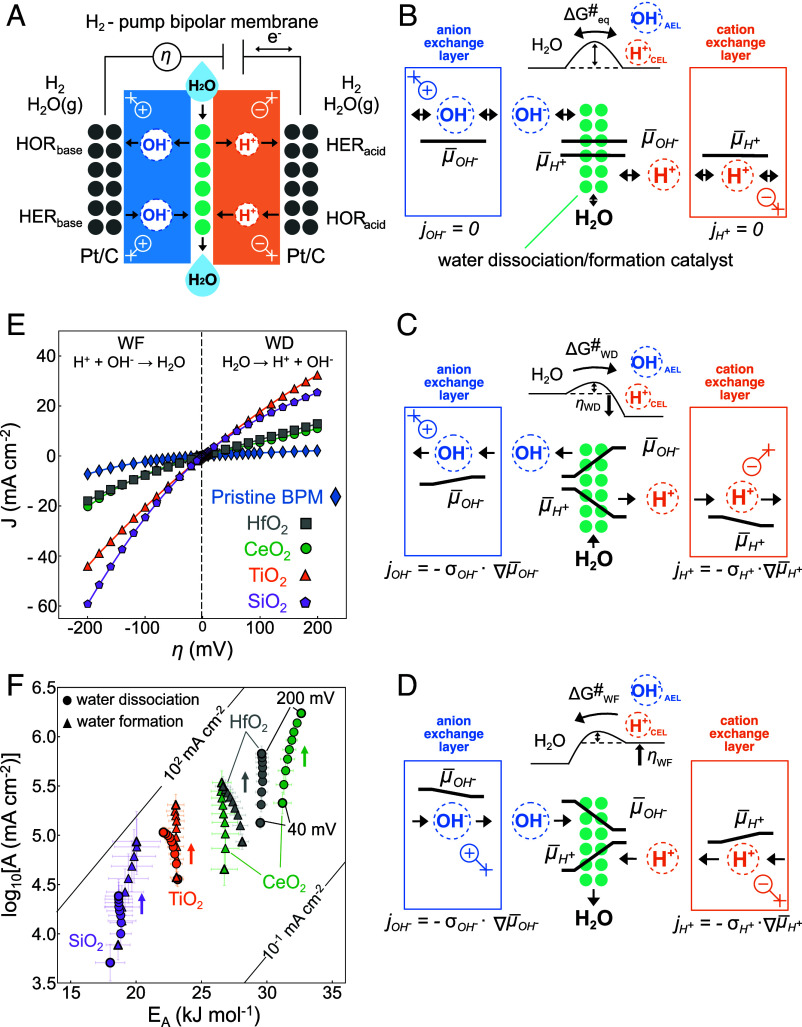
Isolated water dissociation (WD) and formation kinetics in the presence of different metal oxides. (*A*) The H_2_ pump cell uses high mass transport and fast hydrogen evolution (HER) and oxidation kinetics (HOR) in the membranes and high Pt/C loading GDEs, respectively. As a result, the total cell potential directly informs on the WD and formation (WF) kinetics in the spatially isolated bipolar membrane (BPM) junction. The overpotential arises due to the electrochemical potential gradients of the ions. (*B*–*D*) Electrochemical potential profiles of OH^−^ (${\bar{\mu }}_{OH^{-}}$) and H^+^ (${\bar{\mu }}_{H^{+}}$) across the BPM (external electrodes not show) at equilibrium and in WD and WF direction. The activation free energy barrier at equilibrium ${\Delta G}_{\mathrm{eq}}^{\#}$ is changed for WD (${\Delta G}_{\mathrm{WD}}^{\#}$) and WF $\left({\Delta G}_{\mathrm{WF}}^{\#}\right)$ by the applied overpotential in WD (*η_WD_*) or WF (*η_WF_*) direction, respectively. The high conductivity for hydroxides ($\sigma_{OH^{-}}$) and protons ${(\sigma }_{H^{+}})$ in the anion and cation exchange layer, respectively, can support hydroxide ($j_{OH^{-}}$) and proton currents ($j_{H^{+}}$) with negligible gradients in free energy of protons (${\nabla \bar{\mu }}_{H^{+}}$) and hydroxides, ${\nabla \bar{\mu }}_{OH^{-}}$. (*E*) Nonconductive metal oxide nanoparticles can simultaneously catalyze the WD and WF reactions, with varying degrees of reversibility. WD data adapted from our previous study^3^. (*F*) Arrhenius pre-exponential factor (log_10_A) and apparent activation energy (E_A_) for different metal oxides follows the point of zero charge, with more acidic ones having lower E_A_ and A. The iso-current lines are plotted for 65 ºC. Error bars are extracted from Arrhenius linear regressions by fitting 5 currents from experiments at 5 temperatures.

For the WD (H_2_O → H^+^ + OH^−^) and WF (H^+^ + OH^−^ → H_2_O) reactions inside the BPM, a H_2_-pump is used (Fig. 2*A*), where WD and WF are operated at the junction between an acidic CEL and an alkaline anion exchange layer (AEL) (3). In this case, both polymers contact 2 mg cm^−2^ Pt/C GDEs that reversibly operate the HER/HOR and allow us to study the significantly slower WD/WF kinetics in the junction in the presence and absence of electrically nonconductive metal oxide nanoparticles with different points of zero charge (HfO_2_, CeO_2_, TiO_2_, SiO_2_) (3). Compared to the Nafion H_2_-pump, we only study the kinetics at lower current densities (≤~60 mAcm^−2^) to remain near equilibrium and reduce the impact of changing reactant or product concentrations in the BPM junction. We note that the electrodes and membranes support much higher current densities and are not limiting the polarization curves.

At electrochemical equilibrium (Fig. 2*B*), corresponding to a total cell potential of 0 V, the electrochemical potentials of H^+^ and OH^−^ are constant throughout the bipolar junction and cell. Conversely, the Gibbs free energy of the reaction is $\Delta G_{rxn}=0$ and the H^+^ and OH^−^ concentrations, $\left[H^{+}\right]$ and $\left[OH^{-}\right]$, inside the junction (10^−7^ M) are related to the equilibrium constant of water $K_{w}∼\left[H^{+}\right]\left[OH^{-}\right]∼10^{-14}$ at 25 °C. These values arise due to the difference in the free enthalpy between the H_2_O molecule and the solvated H^+^ and OH^−^. At electrochemical equilibrium, the enthalpy of reaction, $\Delta H_{\mathrm{rxn}}$, is fully balanced by the entropy of reaction and the temperature, $T\Delta S_{\mathrm{rxn}}$.

With an externally applied overpotential between the reversible HER/HOR electrodes, electrochemical potential gradients of H^+^ and OH^−^ develop across the ionic junction that tip the reaction out of electrochemical equilibrium, either in WD or WF direction (Fig. 2 *C* and *D*). The high conductivity inside the ion-exchange membranes and the high loading of the HER/HOR GDEs (not shown) require negligible free energy gradients in a certain current density range. In other words, the setup translates the electrically applied overpotential at the electrodes fully ionically into the bipolar junction. Importantly, at heterogenous interfaces, there can be a substantial activation free energy barrier (${\Delta G}^{\#}$) between the H_2_O and solvated H^+^ and OH^−^. The electrochemical potential gradients of H^+^ and OH^−^ that develop across the junction are the origin of the water dissociation (WD) and formation overpotentials, $\eta_{\mathrm{WD}}$ and $\eta_{\mathrm{WF}}$, respectively, which are directly linked to the Gibbs free energy change ($\Delta G_{rxn}=nF\eta$), with n = 1. Note the fact that ion transfer reactions can be described by replacing the free energy of the electron with the free energy of the ion has been shown by others before (36, 54). Whereas abundant evidence shows that the ionic WD reaction can be catalyzed in BPMs (55–60), i.e. ${\Delta G}^{\#}$ is reduced, we only recently related this to the interfacial solvation kinetics of H^+^ and OH^−^ (3). By doing so, we suggested that WD itself is not rate-limiting, but rather the underlying solvation kinetics.

As for the fast HER/HOR on the Pt/c, we treat the WD and formation reactions primarily as *single-step* reactions, i.e. $\alpha_{E}=\alpha_{H}$ and $\alpha_{A}=\alpha_{S}$, because the oxide catalysts remain near their reversible region with a constant apparent activation energy (in a narrow junction with low catalyst loading). To obtain a constant activation energy, any overpotential-dependent coverage terms would have to change in a very specific way when biasing the junction into the opposing WD and WF direction and would quickly lead to unjustifiable overparameterization of the rate equations, if not altogether wrong predictions. However, especially for thicker catalyst layers, the overall water formation and dissociation likely involve surface transport processes across a catalyst bed, as suggested by Boettcher (60). Furthermore, later, we will discuss how the ionic transfer coefficients might relate to a spatially extended profile of the ion electrochemical potential and an ionic multistep sequence.

In the absence of a metal oxide catalyst (Pristine BPM) the polarization curve shows the most pronounced rectification, where the WF currents $j_{\mathrm{WF}}=\left[H^{+}\right]\left[{OH}^{-}\right]k_{\mathrm{WF}}$ in forward direction are larger than the reverse WD current $j_{\mathrm{WD}}=\left[H_{2}O\right]k_{\mathrm{WD}}$ (Fig. 2*E*). For WD, the water concentration is constant for this current density range (61–63), as the membranes and junction are fully hydrated. In contrast, for water formation, the $\left[H^{+}\right]$ and $\left[{OH}^{-}\right]$ remain constant close to equilibrium, as they are controlled by the ion exchange membranes, especially for low junction thicknesses and overpotentials. However, for larger overpotentials, they will start to deviate from the equilibrium concentration. This can be seen for TiO_2_ in Fig. 2*C*, where up to 100 mV, the catalyst increases the WD and WF rates symmetrically and the reactions are run reversibly in cathodic and anodic direction. At higher overpotentials, the WD and WF currents become asymmetric, due to an overpotential induced asymmetry in $k_{\mathrm{WF}}$ and $k_{\mathrm{WD}}$ or because of increasing $\left[H^{+}\right]$ and $\left[{OH}^{-}\right]$ and constant $\left[H_{2}O\right]$. Irrespectively, whereas all polarization curves show a rectification behavior, the metal oxide catalysts clearly increase the steady state rates in WD and in WF direction. Further, whereas the polarization curves at a single temperature (Fig. 2*E*) already indicate a different extent of the reversibility, the temperature dependence is critical to explore the origin of the activity differences.

Fig. 2*F* shows the results of temperature-dependent measurements for each metal oxide catalyst in the range of 40 to 200 mV for both the WD and the WF regimes. For all metal oxides, E_A_ stays approximately constant and the main effect of η is to increase the apparent log_10_A. E_A_ for WD and approximately for WF follow the point of zero charge (PZC) trend of each oxide (CeO_2_ ≈ 8.1 > HfO_2_ ≈ 7.4 > TiO_2_ ≈ 6.8 > SiO_2_ ≈ 2.8) according to common literature values (64). Previously, the general activity of metal oxide nanoparticles was linked to the local pH at the CEL (56), which we refined further when we observed that more acidic particles show a lower E_A_ (3). The relatively large j_0_ values found for these systems (CeO_2_ ≈ 7 < HfO_2_ ≈ 12 < TiO_2_ ≈ 25 < SiO_2_ ≈ 40 mA cm^−2^ at 65 °C) suggest that for the range of current densities studied, the metal oxides are operated in or close to their electrochemically reversible regime, i.e. in an overpotential and current range where the reverse and forward rates contribute to the total current substantially. This is supported by the constant E_A_, indicating that the protonation, and thus coverage and charge, remain constant, as electrically nonconductive oxides are electrostatically decoupled (ϵ≈ 2 to 50) from the junction field. These results are consistent with general conclusions by Hurwitz and Dibiani (65), although their outer-sphere framework did not allow to test for the link between excess charge and activity rigorously.

We observe that oxides with a lower excess charge and lower overpotentials (SiO_2_ and TiO_2_) show similar activation parameters in WD and WF direction, whereas oxides with a higher excess charge and high overpotentials (CeO_2_, HfO_2_) exhibit E_A_ and log_10_A that start to deviate between the WD and WF directions. We ascribe this effect to an interaction between the low activity oxide’s excess charge and the superimposing electric field in the junction that appears due to increasing polarization of the polymer space charge regions (*SI Appendix*, Fig. S5). Finally, we note that to understand the total activity, log_10_A is important. For the oxides in Fig. 2*F*, the lower activation energy was generally related to the higher activity. However, the slightly different activity of TiO_2_ and SiO_2_ (Fig. 2*E*) is not reflected in the comparably large difference in the activation energies (Fig. 2*F*). Therefore, to refine the trends in Fig. 2*F*, a larger range of catalysts with more well-defined PZC values will be needed, including to understand the impact of nonequilibrium pH profiles across extended junctions.

## The Impact of Excess Charge on the Ion Transfer Coefficients

**Fig. 3.**
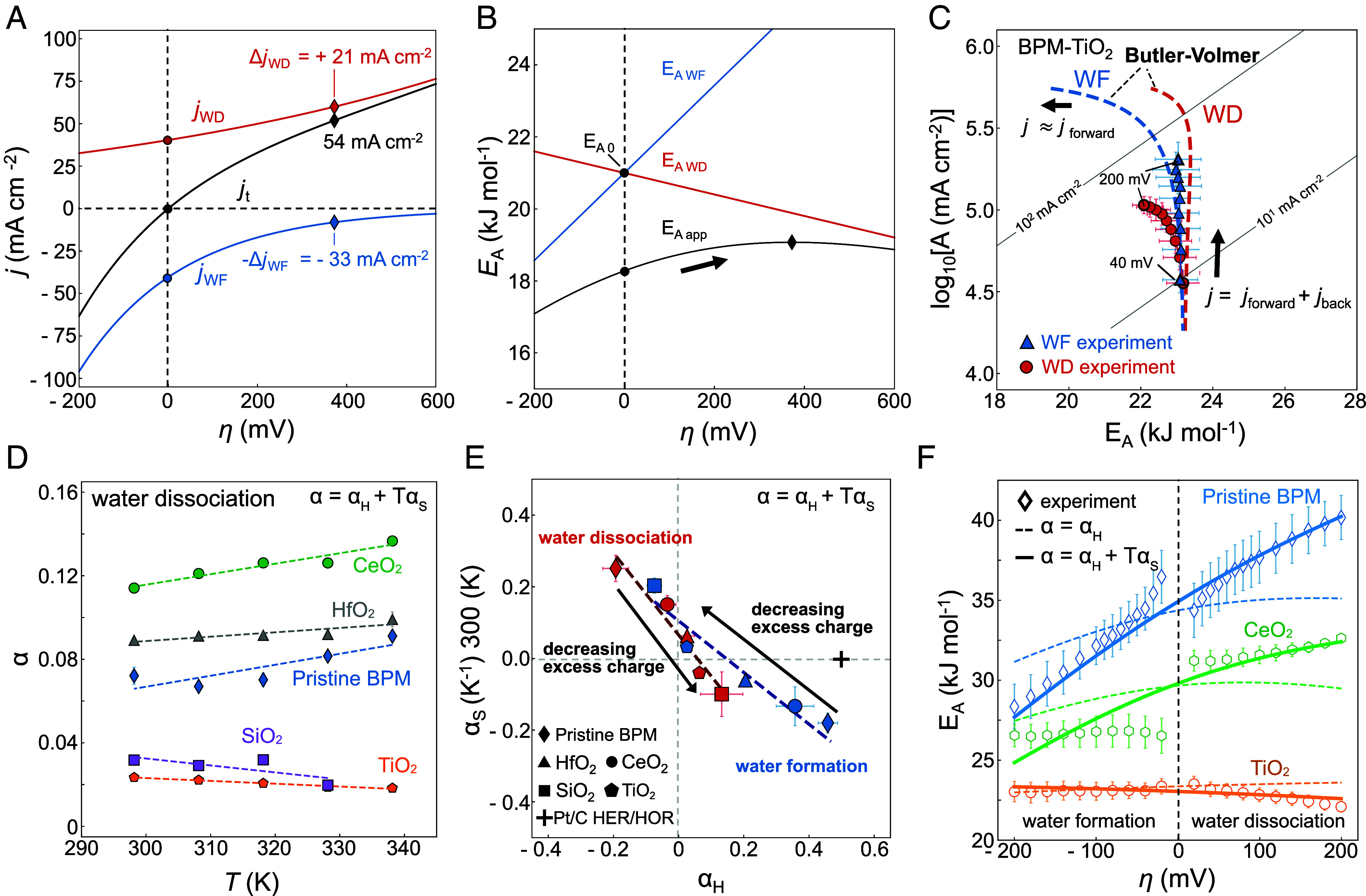
Description of water formation and WD kinetics. (*A* and *B*) Calculation of total and partial current densities ($j_{t},j_{WD},j_{WF}$) at 65 °C for charge transfer coefficients that are different between WD and WF ($\alpha_{\mathrm{WF}}=4\alpha_{\mathrm{WD}}$) and temperature-independent ($\alpha_{S}\approx 0$). For positive WD overpotentials, the net current increases more by the suppression of the WF rate than the actual enhancement of the WD rate ($\left|{\Delta j}_{WD}\right|<\left|{\Delta j}_{WF}\right|$). This leads to an increase of the apparent $E_{A}$, while the individual activation energies for WD and WF decrease. (*C*) Experimental data for a BPM containing 15 µg cm^−2^ of TiO_2_-P25 and a calculation using the fitted results ($\log_{10}A_{0}=5.8$, ${E_{A}}_{0}=26.5\;\mathrm{kJ}\;{\mathrm{mol}}^{-1}$) assuming purely enthalpic transfer coefficients ($\alpha_{WD}=0.04$, and $\alpha_{WF}=0.06$). The apparent pre-exponential factor rises and the activation energy increases slightly for the WD branch as the backward reaction is suppressed. (*D*) Temperature-dependent WD transfer coefficients α. (*E*) Enthalpic ($\alpha_{H}$) and entropic ($\alpha_{S}$) contributions to the total transfer coefficient at 300 K. The values of $\alpha_{S}$ are multiplied by 300 K to show their contribution to the total coefficient at room temperature. For $\alpha_{S}$, see *SI Appendix*, Fig. S7. (*F*) Experimental and fitted $E_{A}$ values using temperature-independent (enthalpic) and temperature-dependent (enthalpic and entropic) charge transfer coefficients.

In Fig. 3*A*, we evaluate whether temperature-independent transfer coefficients ($\alpha =\alpha_{H},\alpha_{S}=0$) may lead in any circumstance to the observed kinetics. We perform this test to evaluate whether more complicated rate equations, with more fitting parameters, are rigorously justified, given the theoretical assumptions and experimental error. Generally, $\alpha_{f}$ and $\alpha_{r}$ are regarded as potential dependence of the forward and reverse rate, respectively. Therefore, if $\alpha_{r}$ is large enough compared to $\alpha_{f}$, the increment of the total current density may be influenced more by the decrease of the reverse reaction rate rather than an increase of the forward reaction. As shown in Fig. 3*B*, for a certain overpotential range into WD direction, this could lead to an increasing apparent activation energy ($E_{A,\mathrm{app}}(\eta )$) (black line) despite a decreasing $E_{A}$ (red dashed lines). $E_{A,app}\left(\eta \right)$ behaves like a skewed hyperbola where individual $E_{A}$ for the reactions are its asymptotes. The shape and extent of $E_{A,app}\left(\eta \right)$ is mainly determined by the magnitude of $j_{0}$ and α. Importantly, the larger $j_{0}$, the larger this “reversible” regime where both the forward and backward reactions influence the total current. Therefore, for a lower $j_{0}$ and/or larger overpotentials, the influence of the reverse reaction is suppressed and the kinetics for the forward current are dominated by the activation parameters of the forward reaction.

In Fig. 3*C* the TiO_2_ BPM WD/WF kinetics (circles and triangles) are compared against predicted Butler–Volmer kinetics with temperature-independent transfer coefficients (dashed lines). As TiO_2_ minimizes excess charge at the active site, the interface becomes highly reversible for the WD/WF reactions. This is reflected in the large $j_{0}$ (~25 mA cm^−2^), leading to an extended reversible current range. Thus, to obtain more insights into such behavior, including for the other oxides, we evaluated the temperature dependence of α. The latter is obtained from a simultaneous fitting of the WD and WF regimes at each temperature while restricting the free parameters, as described in the Methods and consistent with previous studies. The result is shown for WD in Fig. 3*D* (for WF see *SI Appendix*, Fig. S6). In general, less reversible BPMs (pristine BPM, CeO_2_, and HfO_2_) show both higher transfer coefficients and temperature dependencies compared to the reversible BPMs (TiO_2_ and SiO_2_). From the temperature dependence of $\alpha =\alpha_{H}+T\alpha_{S}$ (assuming a single-step reaction) we extract the enthalpic transfer coefficient ($\alpha_{H}$) from interpolation to zero temperature and the entropic coefficient ($\alpha_{S}$) from the experimental temperature range, as shown in Fig. 3*E*.

Fig. 3*F* shows the general ability of the extracted transfer coefficients to describe the overpotential-dependent kinetics, exemplified for E_A_. Starting with the most active catalyst TiO_2_, it shows how slower kinetics require a higher $\alpha_{S}$ for a reasonable match between Arrhenius data and the prediction based on the transfer coefficients. For the intermediate case CeO_2_, the inclusion of $\alpha_{S}$ leads to a better match of $E_{A}\left(\eta \right)$, however, it does not capture the discontinuity in $E_{A}\left(\eta \right)$ between WD and WF. This apparent jump is likely due to the inability of our setup to measure the kinetics at lower overpotentials, where WD and WF activation parameters should merge, and asymmetric solvent polarization differences vanish (*SI Appendix*, Fig. S5). Finally, we emphasize that the transfer coefficients are extracted across an overpotential *range.* Whereas we consider that the water concentration is essentially constant at these low WD current densities, nonequilibrium proton and hydroxide concentrations might impact the obtained transfer coefficients for WF and also lead to a local shift in the pH.

The results in Fig. 3 *E* and *F* hint toward unique aspects of proton and hydroxide charge transfer across the electric field-dependent hydrogen bond network. The charge transfer coefficient can be thought of as the lever of the applied free energy difference and should not be confused with the symmetry factor of the activation free energy barrier (35). For the HER/HOR kinetics in Fig. 1 and *SI Appendix*, Fig. S4, we observed an extended reversible regime ($j_{0}$ ~ 180 mA cm^−2^) with $\alpha_{H}∼0.5$ ($\alpha_{S}∼0$), consistent with a single rate-determining Volmer step dominated by the electron-transfer (35, 36). Electron transfer occurs over very short distances and short times (fs) and the electron transfer coefficient can be assumed either $\alpha_{H}=0.5$ for single steps, or take discrete values depending on the number of electrons transferred before or after a rate-limiting electron transfer step (35). However, for ion transfer across the hydrogen bond network, the transfer coefficient can take values $\alpha_{H}\neq 0.5$ (36).

In Fig. 3*E*, we find that ${|\alpha }_{H}|$ and ${|\alpha }_{S}|$ take small values for the oxides with the highest activities and lower excess charge. A low ion transfer coefficient can explain why for proton-coupled-electron transfer reactions over nonpolarizable surfaces, i.e. in absence of substantial excess charge, such as in Fig. 1, the ion transfer steps are silent and electron-transfer steps dominate the total charge transfer coefficients. In general, a ${|\alpha }_{H}|$ < 0.5 can be understood by a spatial profile of the electrochemical potential of the ion that spreads across the extended hydrogen bond network (35, 36). As a result, the electrochemical potential gradient (the free energy driving force) that acts on the ion at each point along the profile is less sharp and smaller compared to the one for electron transfer over very short distances. In the limit of a charge neutral interface, the ion transfer might become mass transport limited, i.e. the only free energy driving force that is consumed is the one to overcome ionic series resistance. Importantly, in our study here, even the most active catalysts give rise to current densities that are far from mass transport limitation, as the H_2_-BPM setup can support much higher current densities, further supporting the activated nature of the interfacial ion transfer. Finally, in the presence of spatially extended electrochemical potential profiles of H^+^ and OH^−^ across the hydrogen bond network, the question arises whether the ion transfer can even be described as a single-step, instead of a sequence of many small ionic steps, similar to discussions on the metal ion solvation kinetics by Gileadi (36) and ion exchange-mediated (preassociation) steps for supporting electrolytes by Surendranath and team (66).

In Fig. 3*E*, ${|\alpha }_{H}|$ and ${|\alpha }_{S}|$ tend to increase with decreasing activity and increasing excess charge, especially for the CeO_2_ and the pristine BPM, which contains an equilibrium space charge region and electric field, as detailed later. This implies that an increasingly ordered hydrogen bond network systematically decreases the distance over which the electrochemical potential profile spreads, i.e. the latter becomes steeper and the gradient larger. In the limit, the protons and hydroxides might be extracted over very short distances at the surface, even reaching an enthalpic transfer coefficient nearing ${|\alpha }_{H}|∼0.5$. However, regardless whether $\alpha_{H}$ ($\alpha_{S})$ reaches larger values the total α remains relatively low, due to a compensation effect.

Between WD and WF, $\alpha_{H}$ and $\alpha_{S}$ switch signs. For example, for WD, we find $\alpha_{H}<0$ and $\alpha_{S}>0$, and vice versa for WF. This difference between WD and WF, especially for the most charged oxides is related to the shift in the activation parameters observed in Fig. 2*F*, and might be related to the effect of asymmetric polarization effects in WD and WF direction (*SI Appendix*, Fig. S5). Later, we discuss in detail the pristine BPM where the asymmetric polarization is much more apparent. Further, from Eq. **2**, we can see that a negative $\alpha_{H}$ leads to an increasing total activation energy and a positive $\alpha_{S}$ to an increasing pre-exponential factor. This is consistent with the prevalent compensation effect that we observed across many reactions previously (3–7). In fact, later we show explicitly that $\alpha_{H}$ - $\alpha_{S}$ compensation is linked to log A-E_A_-compensation. In contrast, the case of $\alpha_{H}>0$ and $\alpha_{S}<0$ matches the compensation effects reported by Conway (13–16). The compensation has the effect that even high $\alpha_{H}$ and $\alpha_{S}$ values (Fig. 3*E*) can still result in very low total transfer coefficients (Fig. 3*D*), i.e. the compensation is detrimental from an energy conversion standpoint. Importantly, this link between the activation parameters and ion transfer coefficients suggests that apparent entropy-enthalpy compensation is intimately related to the variation of the spatial profiles of the H^+^ and OH^−^ electrochemical potentials with changing water dipole reorientation.

## Irreversible Kinetics at Polarizable Space Charge Regions

**Fig. 4.**
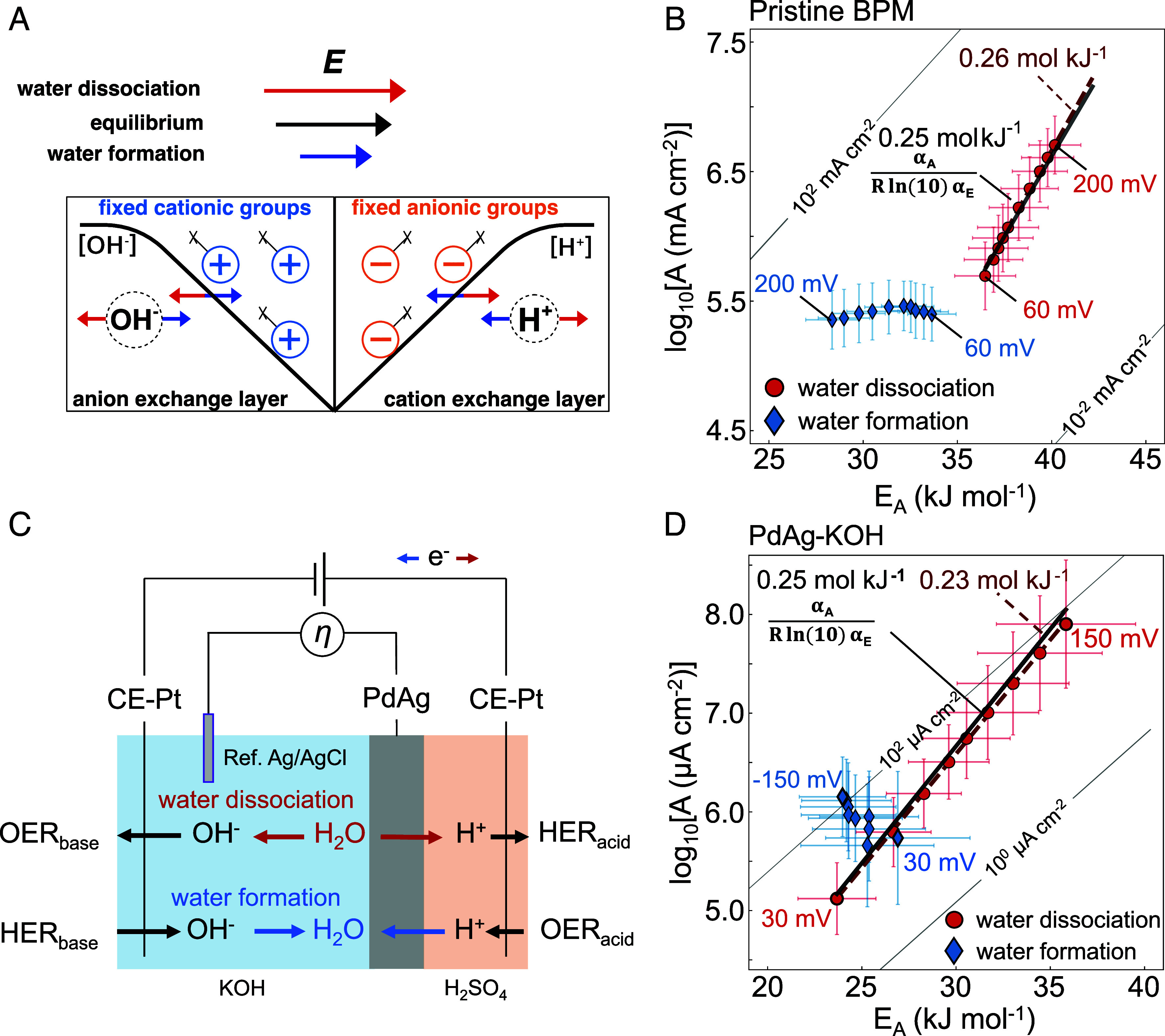
WD and formation (WF) kinetics at bipolar interfaces. (*A*) Schematic of the polarizable space charge region inside the bipolar membrane where the protonation (hydroxylation) state of the fixed anionic (cationic) groups of the anion and cation exchange layers determine the amount of excess charge and, thus, electric field. (*B*) Overpotential-dependent pre-exponential factor, ${log}_{10}A\left(\eta \right)$, and apparent activation energy, $E_{A}\left(\eta \right)$, for the pristine BPM junction. (*C*) Schematics of the four-point probe cell, where WD/WF take place at the interface between the PdAg foil with the KOH electrolyte. (*D*) ${log}_{10}A\left(\eta \right)$ and $E_{A}\left(\eta \right)$ for the PdAg–KOH junction, respectively. For panel *B* and *D* the WD compensation slopes are plotted as dashed red lines (experimental result) and black lines (predicted slope with Eq. **5**).

Pristine BPMs typically display a very low activity and large WD overpotentials even at moderate current densities (Fig. 2). At equilibrium, the interface contains a space-charge region analogous to a p/n-junction. In contrast to active metal oxide catalysts, the reactions now proceed over the fixed anionic or cationic polymer groups of the membranes (Nafion and Piperion) facing the junction. When applying an overpotential into the WD direction, depletion of carriers in the junction can lead to deprotonation and dehydroxylation of the fixed groups. This increases the capacitive excess (space) charge and increases the electric field. In WF direction, protons and hydroxides are injected into the junction and can lead to protonation and hydroxylation, i.e. the space charge region might be reduced (Fig. 4*A*). In fact, previously we correlated overpotential-dependent space charge with the activation parameters (3). We note that the degree of polarization depends on the polymer chemistry and does not have to be universal. Fig. 4*B* shows the kinetic map for WD and WF. Interfacial polarization is not unique to BPMs, but central to metal–solution interfaces (*SI Appendix*, Fig. S1). Thus, to expand the relevance of our findings, we use again a PdAg-H foil setup (67) (Fig. 4*C*), where WD and WF are driven at the interface between liquid KOH and the PdAg foil and the kinetics are studied in isolation (3) (Fig. 4*D*) (see *SI Appendix*, Fig. S8 for the polarization curves).

The comparison of the kinetic maps of the PdAg interface (Fig. 4*D*) reveal very similar kinetics to those of the pristine BPM (Fig. 4*B*). For WF, we observe that the pre-exponential factor is approximately constant, consistent with kinetics that are impacted by a changing rate constant and not a simple increase in [H^+^] and [OH^−^]. Only for the PdAg–KOH interface does the pre-exponential factor slowly rise, indicative of an additional concentration-driven increase in the total WF rate. As we observed previously (3), for WD, compensation effects of similar slope (0.231 mol kJ^−1^ for PdAg and 0.257 mol kJ^−1^ for BPM) are found, where an increasing ${log}_{10}A\left(\eta \right)$ overcompensates an increasing $E_{A}\left(\eta \right)$. The low $j_{0}$ for the pristine BPM and the PdAg–KOH interface suggest that these changes cannot be explained by an impact of the reverse reaction (WF). The same holds for the compensation observed for the HER on Au/C (5), which is irreversible toward the HOR, as dissociative H_2_ adsorption does not occur.

As we detailed before (3–8, 18), ${log}_{10}A$-$E_{A}$-compensation can arise due to overpotential-dependent excess charge or coverage changes, complicating isolation of single rate-limiting steps (68, 69). Therefore, in the following we use Eq. **3** and discuss both aspects. Since $\alpha_{E}$ and $\alpha_{A}$ modulate the overpotential dependence of ${log}_{10}A$ and $E_{A}$, their relative magnitude also informs on the compensation. We derive a linear relationship (*SI Appendix*, Supplementary Note 2) between ${log}_{10}A\left(\eta \right)$ and $E_{A}\left(\eta \right)$, where the compensation slope ($b_{\mathrm{CE}}$) is

[5] $$b_{\mathrm{CE}}=-\frac{\alpha_{A}(\eta )}{\ln\left(10\right)\;R\;\alpha_{E}(\eta )},$$

with inverse activation energy units (mol kJ^−1^) and intuitively informing on the ratio between changing logA by changing $E_{A}$.

Fig. 4 *B* and *D* show a very good match between the value obtained via the charge transfer coefficients (Eq. **5**) and the one obtained from overpotential-dependent Arrhenius analysis. Further, we obtain excellent agreement between the methods for the compensation slopes (~0.31 mol kJ^−1^ and ~0.32 mol kJ^−1^) when applying the same method to our previous results on the acidic HER on Au (5) (*SI Appendix*, Fig. S9). Last, we applied Eq. **5** to the temperature-dependent gradient variation of (η – ${log}_{10}j$), obtaining apparent Tafel slopes and ion transfer coefficients that predict the differential compensation slopes $b_{CE}$ for the points in a kinetic map (*SI Appendix*, Fig. S10). This consistency further highlights the quality of the dataset. For example, the pre-exponential factor is obtained from Arrhenius analysis via interpolation to infinite temperature, but the respective ion transfer coefficient stems from the slope in the experimental temperature range (see also *SI Appendix*, Fig. S9).

Eq. **5** shows that a common compensation region can result from overpotential-dependent $\alpha_{A}\left(\eta \right)$ and $\alpha_{E}\left(\eta \right)$ and nonlinear Tafel slopes (see also the analysis of the Au/C in *SI Appendix*, Figs. S9–S10). For the BPM, we assign these overpotential Tafel slopes and ion transfer coefficients even at higher overpotential primarily to the impact of the electric field on the activated ion transfer. The focus on the electric field in this study is due to the results obtained for various metal oxides in their electrochemically reversible regime where overpotential-dependent coverage terms and multistep sequences can be neglected. Further, the activation parameters and ion transfer coefficients depend on the excess charge, the transfer coefficients take values distinctly different from electron-transfer limited kinetics, they display compensation effects, and they change asymmetrically in forward WF vs. reverse WD direction, especially for the pristine BPM with an in intrinsic overpotential asymmetry of the electric field inside space charge region. These results are difficult to reconcile with a sole coverage dependence of the activation parameters over an otherwise neutral surface or by considering only surface pKa values and thermodynamic arguments from bulk chemistry, which neglect the interfacial electric field. However, the overpotential-dependent space charge region might at least partially be understood by a coverage-continuum of activation parameters for many sequential steps with a different hydroxylation and protonation state.

## Discussion

Since the invention of the BPM in 1956 by Frilette (70), BPM research and development were challenged by the buried nature of the junction and complex co- and counter-ion effects in electrodialysis environments (71, 72). Across the literature, macroscopic polarization curves are regularly fitted with overparameterized rate equations speculating about intricate physics. The activity of interfacial water formation was never understood, because in many electrodialysis conditions salt ions (e.g. K^+^ and Cl^−^) are injected into the junction and typically remain fully dissociated and solvated, i.e. the reaction is limited by mass transport into and out of the junction. However, for weak acids or bases, it was shown that ions can block the junction and a larger applied cell potential is needed to exceed a new equilibrium potential (73, 74). Due to such complications, others and we transferred the BPM into a pure water environment (56, 63, 75), which enabled enormous advancements in understanding and practical developments (3, 23, 58–60).

Here, electrochemical Arrhenius analysis in the reversible regime rigorously reduces the free parameter space to a minimum. This provides the most comprehensive BPM kinetic picture to date that naturally includes the water formation kinetics and removes the artificial separation of electric-field and catalyst effects that is dominating the BPM literature. Collectively, our results (3–7) shift the focus away from the notion that WD or WF themselves are limiting, to the question of how the polarization of the interphase impacts the proton and hydroxide solvation. With that, our results join BPMs with fundamental electrochemical concepts more broadly and also highlight the need to resolve the role of excess charge with more fidelity, especially in the presence of spatially extended pH-profiles for extended junction thicknesses.

Beyond BPMs, our results are important to electro-, bio-, and even geochemistry where interfacial solvation is involved in many reactions. In fact, all acid–base reactions in polar media, designated by vertical lines in Pourbaix diagrams, involve ion solvation. Further, interfacial ion solvation and intercalation is omnipresent in batteries. Our results imply that such ionic reactions possess free energy barriers that are linked to the amount of excess charge at the interface. This has also important ramifications for electrocatalyst activity.

**Fig. 5.**
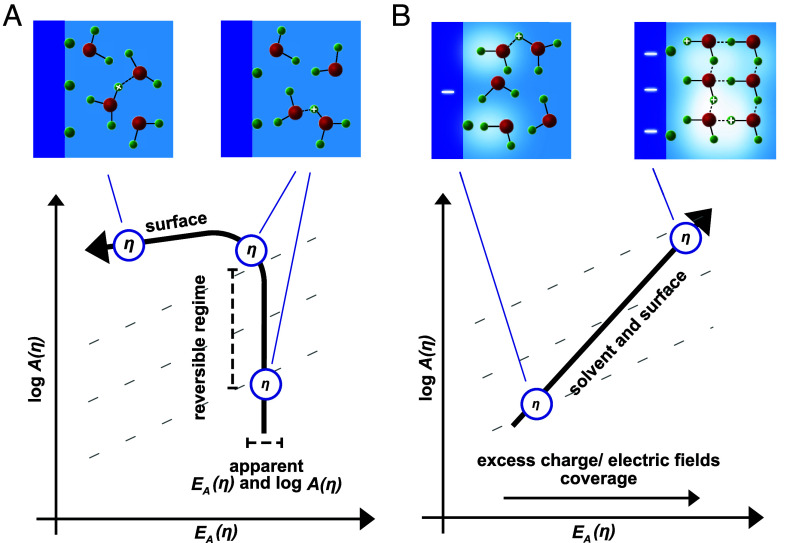
The impact of excess charge on the reversibility of interfacial ion solvation. (*A*) Potential-dependent ${log}_{10}A\left(\eta \right)$ and $E_{A}$ for a nonpolarizable interface. At low overpotentials, the current density is similar to $j_{0}$ and the system is in its reversible regime. At higher overpotentials, the influence of the reverse reaction is suppressed and the kinetics show decreasing $E_{A}$. (*B*) Potential dependent ${log}_{10}A\left(\eta \right)$ and compensating $E_{A}\left(\eta \right)$ for irreversible kinetics on polarizable interfaces. Starting at low overpotentials, the interfacial excess charge and the electric fields might impact the activation entropy and enthalpy of the interfacial solvation step and, together with the overpotential-dependent coverage terms, induce multistep sequences at higher overpotentials.

Nonpolarizable interfaces can display almost ideal outer-sphere kinetics (Fig. 5*A*). However, in general, these are rather unique conditions, such as for the fast kinetics for the HER/HOR on the Pt group metals or Ag^+^ deposition/corrosion. Even the metal oxide catalysts in bipolar membranes generally contain remaining changes in the pre-exponential factor and compensation effects that are related to the excess charge. In fact, most reactions we studied display compensation effects at lower overpotentials between an increasing pre-exponential factor and increasing activation energy (Fig. 5*B*) (3–7). Excess charge and coverage changes can emerge whenever an (electro)chemical overpotential is applied to an interface, but electronic or ionic charge transfer is impeded by an activation free energy barrier. In this context, it is important to note that an interfacial barrier for ion (de)solvation is the prerequisite for the emergence of electronic excess change on the surface (3, 4, 6), because the former hinders the formation of charge-compensated bonds. The interfacial solvation kinetics, thus, become an intimate component of the kinetic overpotential for polarizable interfaces. In fact, our results show that the degree of reversibility and the suppression of the reverse rate are fundamentally related to the emergence of such compensation effects. This provides a substantial step forward in understanding compared to frameworks that relate the reversibility and activity of a reaction solely to the magnitude of a static exchange current density.

Finally, we often observed an overpotential where the kinetics after the compensation region appear to transition into an apparent Butler–Volmer region (5–7) and reasoned that this could happen after a saturating ordering inside the hydrogen bond network, i.e. that in the case of electric-field induced water ordering, a sequential single-step picture might be viable, which recently found theoretical support, as we detail below (76). Regardless, to understand transitions between kinetic regimes in detail, the overpotential-dependent coverage terms and overpotential-dependent rate-limiting steps need to be considered, emphasizing the need for microkinetic models (18, 19, 68, 69).

## Conclusions

In the bulk, water formation $\left(H^{+}+OH^{-}\to H_{2}O\right)$ is one of the fastest reactions known to mankind and generally considered to be mass transport limited by rapid Grotthuss transport with a low $E_{a}^{\mathrm{WF}}$ of around (77) ~15 kJ mol^−1^. The activation energy of WD ($71\;\mathrm{kj}\;{\mathrm{mol}}^{-1}$) was traditionally estimated (60, 78) *via* the standard enthalpy $\Delta H^{\circ }=E_{a}^{\mathrm{WD}}-E_{a}^{\mathrm{WF}}∼56\;\mathrm{kj}\;{\mathrm{mol}}^{-1}$. In contrast, here we move this fundamental reaction to an interface where we directly control the reaction free energy $\Delta G_{\mathrm{rxn}}$ *via* the electrochemical potentials of the protons and hydroxide species. This allows us to move the reaction out of electrochemical equilibrium ($\Delta G_{\mathrm{rxn}}=0$), either into the WD $\left(\Delta G_{\mathrm{rxn}}>0\right)$ or WF $\left(\Delta G_{\mathrm{rxn}}<0\right)$ direction. We find that the activation free energy barriers cannot be solely understood by only considering the activation energies. Instead, we find logA-E_A_-compensation inside the hydrogen bond network. The activation energies and pre-exponential factors for WF and WD increase with equilibrium excess charge and electric fields, but remain identical close to electrochemical equilibrium. However, at applied overpotential, we find that asymmetric build-up of excess charge and electric fields lead to different activation parameters, which is most pronounced for the pristine BPM. Noteworthy, bulk WD is assumed to take place due to strong electric field fluctuations (29). Thus, keeping the different water structure in the bulk in mind, our results might help us to understand why the activation energy of bulk WD is so large and why it differs so much from bulk WF.

Our results suggest that electric fields modulate the spatial profile of the ion electrochemical potential across the hydrogen bond network. We find ionic transfer coefficients that might arise due to a collective reaction sequence consisting of many small steps for charge neutral conditions that, however, collapses into a sharply defined reaction zone in the presence of strong electric fields. Clearly, advanced molecular dynamics (MD) simulations at charged interfaces will be critical to test this picture. Noteworthy, recently Litman et al. (79) used ab initio MD to show that externally applied electric fields can dramatically enhance reactivation rates due to electric-field-dependent entropic changes and neural networks started to capture long-range electrostatic interactions for externally applied fields for the WD reaction on metal oxide surfaces (33). Further, using machine learning potentials and grand canonical density functional theory, Tian et al. (76) showed that the electric-field-dependent ordering of the hydrogen bond network not only facilitates proton and hydroxide transfer, but can even dominate the free energy changes of the total electrocatalytic reaction over polarizable electrodes, in strong support of our hypotheses provided here and previously (3–7). These results motivate to combine interfacial solvent spectroscopy (26, 31) and new theory (33, 76, 80–82) with electrochemical Arrhenius analysis to gain not only molecular-level insights, but to understand their impact on practically relevant processes.

## Methods

The experimental methods are similar to the ones previously reported by our group in Ref. 3 and detailed in the *SI Appendix*, *Methods*. Further, temperature-dependent currents were not only evaluated with electrochemical Arrhenius analysis but also by studying the temperature dependence of the overpotential dependence of the rate equations, as quantified by the general charge transfer coefficient $\alpha \left(\eta ,T\right)$. To that end, a constrained fitting routine was employed, consistent with previous studies (42, 43) and which also allowed to calculate the kinetics from the transfer coefficient and show the consistency with the electrochemical Arrhenius analysis. More details can be found in the *SI Appendix*, *Methods*.

## Supplementary Material

Appendix 01 (PDF)

## Data Availability

All study data are included in the article and/or *SI Appendix*. All raw data are available in the Zenodo repository under DOI: 10.5281/zenodo.19347490 (83).
