# Supplementary material for: The role of interfacial excess charge in the reversibility of proton and hydroxide solvation in electrocatalysis and bipolar membranes
Source: Proc Natl Acad Sci U S A. 2026 Apr 16;123(16):e2531938123. doi: 10.1073/pnas.2531938123 (PMC13099558; doi:10.1073/pnas.2531938123)
Supplement: Supplementary file 1 — Appendix 01 (PDF) [file pnas.2531938123.sapp.pdf]

The PDF file includes:

Supplementary Notes 1-2

Supplementary Figures 1-11

Supplementary References

Supplementary Methods

Membrane and Ionomers

Nafion 212 (Fuel Cell Store) (~ 50 μm) was used as the cation-exchange layer (CEL). The as-received membranes were cut into 1.5x1.5 cm^2 pieces and immersed for at least 24 h in deionized water before further use. PiperION-A25-HCO3 (~25 μm thickness) and PiperION-A20-HCO3 (~20 μm thickness) (Versogen) were used as anion exchange layers (AEL). The as-received membranes were cut into 1.5x1.5 cm^2 pieces and immersed in 0.5 M KOH for at least 2 h after which the KOH was renewed, leaving the pieces in the solution for at least another 24 hours. Nafion 520 (Sigma Aldrich) was used as an ionomer to prepare the electrodes for the acidic experiments with the CEL and a dispersion of PiperION-A25-HCO3 in ethanol (Versogen) was used as the ionomer for the experiments in base using an AEL.

Electrode Preparation

The gas diffusion electrodes (GDEs) were fabricated by spray-coating a dispersion containing Pt/C and respective ionomers onto Freudenberg H23C2 (Fuel Cell Store), a standard carbon paper support with microporous layer and hydrophobic treatment widely used for polymer fuel electrolyte fuel cells. High-loading counter electrodes were prepared by mixing 100-150 mg of Pt/C 70% and 400-600 mg of the respective ionomer solution at 5 % wt with 0.5 g of H_2O and 1.7 g of isopropyl alcohol (IPA). These high-loading electrodes were used as counter electrodes for the Pt HER/HOR experiments and for the BPM WD/WF experiments. The electrodes facing the CEL were prepared with Nafion 520 as the ionomer and the ones facing the AEL were prepared with PiperION-A25-HCO3. Variable-loading working electrodes for the Pt HER/HOR MEA experiments were prepared by mixing different amounts of Pt/C 70% with Nafion 520 ionomer dispersion and the same amount of H_2O and IPA as the high-loading electrodes. The dry ionomer for all electrodes was of 20 % wt. The dispersions were sonicated for at least 4 hours before spraying onto Freudenberg H23C2 cut to ~ 5x5 cm^2 and heated at 85 ºC. The resulting total loading for the Pt/C counter electrodes ranged between 1.5 ~ 2 mg cm^{-2}. Finally, the GDEs were cut into 1x1 cm^2 pieces to be used in the electrochemical setup.

Membrane Electrode Assembly

The acidic Pt HER/HOR and BPM WD/WF electrochemical experiments were performed in a regular membrane-electrode assembly (MEA). For the acidic Pt HER/HOR experiments, a high loading Pt/C counter electrode with Nafion ionomer was placed inside the gasketing space composed of two gaskets of 150 and 50 μm. Next, an ion-exchanged Nafion 212 membrane was placed on top of the GDE, making sure that the electrode area is fully covered. Another two gaskets (150 and 50 μm) were placed on top before finally adding a variable-loading Pt/C working electrode facing the membrane. Similarly, for the BPMs, a high-loading Pt/C-PiperION electrode was placed in a 250 μm gasket and covered with a PiperION-A25 AEL. The AELs were thoroughly washed and soaked in pure water before being placed on top of the electrode. Next, a pristine or a metal oxide coated Nafion 212 CEL was placed on top of the AEL, ensuring that for the metal oxide-covered membranes, the coating faced the AEL so that the metal oxide was placed within the BPM junction. Finally, another 250 μm gasket and a high-loading Pt/C-Nafion electrode were placed with the electrode facing the CEL. For both experiments, the whole stack was fixed with 8 screws with a torque at 4 NM. The MEA setup

provides continuous physical compression between all layers during operation and enables a zero-gap contact between GDEs and ionically conductive membranes. This allows us to operate the MEA without liquid electrolytes, feeding only a pure-water humidified gas stream of H_2.

Electrochemical Setup and Initial Stabilization

The electrochemical experiments were performed using a Gamry® Reference 3000 potentiostat with a fuel cell humidification system (Fuel Cell Technologies Inc, Model LFHS-C) that controlled the MEA cell temperature, as well as the humidification, flow rate, back-pressure and temperature of the H_2 feed. Humidified H_2 gas was fed to both, the counter and working electrodes (anode and cathode), operating the cell as a H_2 pump. Due to the fast kinetics of the acid HOR (HER) in the highly loaded (1.5 ~ 2 mg cm^{-2}) Pt/C counter electrode GDEs, the cell potential informs directly on the HER (HOR) kinetics for the lower loadings (4-23 µg cm^{-2}) in the working electrode. Similarly, for the BPM, due to the fast acid and alkaline HER/HOR kinetics at the electrode-membrane interfaces, the cell potential informs directly on the WD/WF kinetics spatially isolated at the BPM junction.

For both types of experiments, humidified H_2 gas heated to 65 ºC was fed into the MEA cell through heated inlets at 70 ºC (to prevent condensation) and at equal flow rates of 25 standard cubic centimeters per minute (SCCM) until a backpressure of 1 bar was obtained for both the counter and working electrode chambers. Then, the cell was heated from room temperature to 63.5 ºC while applying a reductive potential in the working electrode (Pt HER/HOR experiments) or 200 mV of WF potential (BPM experiments) to keep track of the current evolution during the heating process for a minimum of 40 min as this showed to improve the stability of the measurements.

Chronoamperometric and Impedance Temperature Measurements

The electrochemical kinetics of the MEA experiments were evaluated by chronoamperometric measurements fixing the potential and measuring the current in steps that lasted from 30 s to 3 min to ensure stable performance. The last 15 points of each step were taken to calculate the average current and potential. For the metal oxide BPM experiments, the sequence of measurements always began from WD to WF, as higher reverse WD bias showed larger stability. Nonetheless, even in WF direction, we did not observe any significant variation in the performance over the measurement time (Supplementary Figure 11), suggesting the absence of significant metal oxide dissolution effects, catalyst reconstruction, or degradation during the measurement. For the pristine BPM, the absence of metal oxide allowed us to measure the system from the WF to the WD (Supplementary Figure 11). We note, that the absence of substantial dissolution effects in our study operated at lower current densities and with thin junction thicknesses does not imply that metal oxides are generally stable in BPMs. With increasing current densities, especially in WF directions as H^+ and OH^- are injected into the junction, non-equilibrium pH-profiles can be established across the junction that could trigger overpotential dependent dissolution effects. This appears particularly relevant for larger junction thicknesses and catalysts beds, as the pH could not be controlled by the ion-exchange capacity of the adjacent polymer membranes. To understand such effects, extended stability studies at higher current densities and also junction thicknesses (that might initially support higher total performance) will be needed in the future.

The measurements were repeated in the temperature range between 65 and 25 ºC by setting the nominal MEA cell temperature at 63.5, 53.5, 43.5, 33.5, and 25.0 ºC. The humidified H_2 gas was supplied to both electrode chambers at 65, 55, 45, 35, and 25 ºC, and the anticondensation inlets were kept 5 ºC hotter at 70, 60, 50, 40 and 30 ºC. The nominal MEA cell temperatures were kept slightly lower than the H_2 gas to avoid drying of the membranes.

For the Pt/C HER/HOR experiments, after each chronoamperometry potentiostatic electrochemical impedance spectroscopy was performed in the range of 1-10^6 Hz with a voltage amplitude of ± 10 mV. The solution resistance for each experiment was obtained by extracting the real impedance at high frequencies, where the imaginary component is the closest to zero. For the BPM measurements, only a subset of the measured potentials was selected to measure the impedance response.

PdAg Experiments

The PdAg (75:25) foils (Fisher Scientific) were cleaned in a 1:2 solution of H_2O_2:HNO_3 for 15 min, followed by a thorough rinse in pure water. Then the foils were mounted into a custom plastic (HDPE) H-type cell with electronic leads made from titanium. A leak-less Ag/AgCl (eDAQ) reference electrode was used, together with a Pt counter electrode (separated with a Piperion membrane) in the analytic chamber. A second Pt counter electrode was used in the auxiliary compartment. The analytic chamber (with the reference electrode) was filled with 0.1 M KOH (Fisher Scientific, semiconductor grade) and sparged with Ar to remove O_2. The auxiliary chamber was filled with 0.1 M H_2SO_4 (99.99%). The foil area was taken as the one defined by the two o-rings separating the compartments. Before measurements, the metal membrane was charged for 10 min with a hydrogen evolution reaction (HER) current of 200 mA cm^{-2} within the analytic chamber. Afterwards, bubbles were removed from the foil surface with a plastic pipette. Current was driven between the two counter electrodes and the potential of the PdAg membrane was measured vs the Ag/AgCl electrode. By subtracting the equilibrium open-circuit potential from the measured potential, the overpotential for the interfacial WD and WF reactions can be extracted. For more details, the reader is referred to the recent publication by Tang et al.[1]. For the temperature-dependent measurements, the whole H-cell was placed into a separately heated temperature bath. To determine the WD/WF overpotential directly, chronopotentiometries were performed as we observed a small shift of the open-circuit potential, which prevented us from performing comparative chronoamperometric measurements.

Temperature Dependent Analysis

The overpotential-dependent pre-exponential factor $A(\eta)$ and activation energy $E_A(\eta)$ are extracted by fitting the temperature-dependent current densities j to the linear form of Arrhenius equation:

$$\log_{10} j = \log_{10} A(\eta) - \frac{\mathrm{E_A}(\eta)}{\ln 10\, R} \frac{1}{\mathrm{T}} \qquad \mathbf{(1)}$$

Where $\log_{10} A(\eta)$ and $E_A(\eta)$ are obtained from the origin intercept and slope of a linear regression of $\log_{10} j$ vs T^{-1}. The standard errors obtained from the slope and origin intercept are plotted as error bars for $\mathrm{E_A}(\eta)$ and $\log_{10} A(\eta)$, respectively. Note that the vibrational frequency term $(k_b\mathrm{T}/h)$ inside the pre-exponential factor makes it temperature-dependent and should lead to a non-linear dependence of $\log_{10} j$ vs T^{-1}. However, its effect in the temperature

range accessible to experiments in aqueous solutions is limited. Alternatively, one can use the Eyring-Evans-Polanyi formalism, dividing the experimental current density by the temperature, and then performing a linear fit.

Fitting Electrochemical Data

The transfer coefficients α and exchange current densities j_0 were extracted for each temperature by performing a least-squares fitting of the total current density (j):

$$j = j_a - j_c = j_0 \left[\exp\left(\frac{\alpha_a F\eta}{RT}\right) - \exp\left(\frac{-\alpha_c F\eta}{RT}\right)\right] \quad (2)$$

consistent with previous studies[2]. This approach is only possible when measuring both the cathodic (j_c) and anodic (j_a) current densities. Often, this is not possible, and Tafel analysis is performed, measuring j at high overpotentials to suppress the influence of the reverse reaction and fitting η vs $\log_{10} j$. By convention, we assign the anodic transfer coefficients (α_a) to HOR and WD and the cathodic transfer coefficients (α_c) to the HER and WF reactions.

Electrochemical systems exhibiting high j_0 values often times result in linear I-V polarization curves which are hard to fit unambiguously with general least-squares algorithms. The reason behind this is that a linear I-V response can be obtained by virtually an infinite number of linear combinations of exponentials with a very high amplitude (given by j_0). As such, these approaches tend to minimize the error gradient by overestimating j_0 in relation to α, and as such, they tend to yield physically unrealistic values. To avoid this, first we obtain a reasonable estimate of j_0 from the micro-polarization region (± 10 mV), assuming that j behaves linearly for small η consistent with previous studies[2]. In this region, the applied overpotential does not substantially displace the system from the electrochemical equilibrium, probing the linear current response dominated by the exchange current density (j_0) of the reactions taking place at the interface:

$$j = \frac{(\alpha_a + \alpha_c) j_0 F}{RT} \eta \quad (3)$$

The slope of this expression informs on $(\alpha_a + \alpha_c) j_0$, which carries the problem of imposing the value of the sum of the charge transfer coefficients. However, by performing the linear Arrhenius analysis of $(\alpha_a + \alpha_c) j_0$, one can estimate E_{A_0} approximating the sum of transfer coefficients to be constant with temperature to avoid non-linearities in the fit. Then, E_{A_0} is used as a fitting constrain to screen the $\log_{10} A_0$ values that minimize the least-squares error, finally obtaining α_a, α_c, E_{A_0}, $\log_{10} A_0$ (and implicitly j_0).

Calculating Kinetic Maps based in transfer coefficients.

The potential-dependent $E_A(\eta)$ and $\log_{10} A(\eta)$ were calculated by inserting different values of α_a, α_c, and j_0 into **equation (2)** to predict current densities at different temperatures and then using **equation (1)** to perform the linear Arrhenius analysis of the current densities calculated for different overpotentials. At each temperature, j_0 was calculated using E_{A_0}, $\log_{10} A_0$ and the transfer coefficient for each reaction was calculated using equations (2-3) in the main. The approach of calculating $\alpha(T)$ for each temperature is identical to using the explicit potential dependence of $\log A$ and E_A by using α_A and α_E as introduced in equation (3).

Supplementary Note 1

For an activated electrochemical process, the free energy of activation, $\Delta G^{\#}$, is assumed to vary linearly with the applied overpotential, η:

$$\Delta G^{\#} = \Delta G_0^{\#} - z\,\beta\,F\,\eta = \Delta H_0^{\#} - T\Delta S_0^{\#} - z\,\beta\,F\,\eta \quad (4)$$

Where $\Delta G_0^{\#}$, $\Delta H_0^{\#}$ (or E_{A_0}), and $\Delta S_0^{\#}$ are the standard Gibbs free energy, enthalpy (or energy), and entropy of activation at electrochemical equilibrium, respectively. The parameter z is the charge valence (assumed 1 for the net transfer of one electron charge), β the barrier's symmetry coefficient, F Faraday's constant, and T the temperature. The symmetry coefficient β of the activation free energy barrier and is defined for a *single* rate-limiting step, with a value between 0 and 1. However, experimentally, one can only access the charge transfer coefficient $\alpha(T,\eta)$[3], which informs on the general overpotential dependence for any single- or multi-step reaction and can be overpotential and temperature dependent and reach any value[4]. In general, the extraction of β requires more unique conditions[4], such as knowledge of the exact free energy gradient at the interface. Thus, we use α even when discussing assumed single-step ion transfer.

The temperature dependence of $\alpha(T,\eta)$ was observed as early as 1947 by Agar[5] and studied extensively by Conway[6–9] for the HER on the sp-metals. For these metals, he assumed that the overpotential dependent kinetics inform on one rate-limiting step, i.e. that overpotential dependent coverages that could induce changing rate-limiting steps are negligible. This was based on the observation of linear Tafel slopes ($b = \ln 10\,RT/(F\,\alpha(\eta,T))$) over *decades* of current densities. Therefore, to capture the temperature dependence he introduced a linear dependency of α (or β) with T:

$$\alpha(T) = \alpha_H + \alpha_S\,T \quad (5)$$

Where α_H and α_S (K^{-1}) are the enthalpic and entropic components, respectively.

The experimentally accessible total current density, $j = j_c - j_a$, is composed of the cathodic, j_c, and anodic, j_a, current densities. Depending on the activity, at low overpotentials the reverse and forward rates can be substantial and higher overpotentials are needed to suppress the reverse rate. The anodic current density for a single-step reaction is:

$$j_a = A(\eta)\exp\left(\frac{-E_A(\eta)}{RT}\right) = \left[n\,F\,c_i\,v\exp\left(\frac{\Delta S_0^{\#} + \alpha_S\,F\eta}{R}\right)\right]\exp\left(\frac{-E_{A_0} + \alpha_H\,F\eta}{RT}\right) \quad (6)$$

The Arrhenius pre-exponential factor, $A(\eta)$, contains the activation entropy, $\Delta S^{\#}(\eta)$, the number of electrons passed in the reaction, n, Faraday's constant, F, and the concentration of reactant i, c_i, and the ideal gas constant, R. We note that the frequency factor, v, can also be grouped into the exponent as vibrational entropy[10]. For a cathodic process, the equation has to be written with the signs of α_S and α_H inverted.

The above equations give room for entropic changes *via* the temperature dependence, but explicitly assume a single rate-limiting step. At higher current densities, this single-step manifests itself in linear Tafel slopes, $b = \ln 10\,RT/(F\,\alpha(\eta,T)$, i.e. $\alpha(\eta,T) = \alpha(T)$. Historically, the observation of constant Tafel slopes over *decades* of currents suggested a *single* rate-limiting step[3,4,6,11]. Whereas this reasoning might be applicable for single electron transfer steps, for activated ionic processes across the double layer the focus on constant Tafel slopes

is not warranted, as we discuss later. Regardless, the need for high overpotentials renders the analysis of constant Tafel slopes unsuitable in the study of kinetic limitations that arise at low overpotentials, especially for fast reactions with an appreciable reverse rate.

Beyond single-step kinetics, in general, the charge transfer coefficient, $\alpha(\eta, T)$ and, thus, the Tafel slope, $b(\eta, T)$, might be overpotential dependent due to an underlying multi-step reaction. Therefore, in all generality, the transfer coefficient can be split into

$$\alpha(T, \eta) = \alpha_E(\eta) + \alpha_A(\eta)\, T \quad \textbf{(7)}$$

with an overpotential dependent component for the apparent activation energy, α_E, and Arrhenius pre-factor, α_A. Consequently, while we treat the kinetics primarily as single-step kinetics in this study, we will discuss the possibility of multi-step kinetics for ion transfer through the extended hydrogen bond network and overpotential dependent space charge.

Supplementary Note 2

Derivation of the compensation slope from the charge transfer coefficients

For a given electrochemical system with a reversible cathodic and anodic reaction, e.g., the hydrogen evolution and oxidation reactions, the total current density (j) is the difference between the anodic (j_A) and cathodic partial current density. For sufficiently large anodic (cathodic) overpotentials (j_B) the influence of the cathodic (anodic) reaction is suppressed:

$$j = j_A - j_B \approx j_A \quad \textbf{(8)}$$

The potential dependence for this current density simplifies to the one of just the anodic rate:

$$j = j_0 \exp\left(\frac{\beta\, z\, F\, \eta}{RT}\right) = n\, F\, c_i\, k_0 \exp\left(\frac{\beta\, z\, F\, \eta}{RT}\right) \quad \textbf{(9)}$$

Where j_0 is the exchange current density, β the symmetry coefficient, z the charge valence of the species involved in the reaction, F is Faraday's constant, η is the overpotential, R is the ideal gas constant, T is the temperature, n is the number of electrons transferred, c_i is the product of the concentration of the i-reacting species and k_0 is the rate constant for the electrochemical reaction. Note that this expression is written specifically for the anodic rate but that can be applied to the cathodic rate if the sign of the current and the symmetry coefficient is inverted. The rate constant k_0, can be further expanded to include explicitly the equilibrium pre-exponential factor A_0 (which informs on the entropy of activation of the reaction $\Delta S_0^{\#}$) and the activation energy $\mathrm{E_{A_0}}$ (related to the activation enthalpy $\Delta H^{\#} = E_A - RT$):

$$J = A_0 \exp\left(\frac{-\mathrm{E_{A_0}}}{RT}\right) \exp\left(\frac{\beta\, z\, F\, \eta}{RT}\right) \quad \textbf{(10)}$$

Note that A_0 has the units of current density ($A\ m^{-2}$) and includes c_i, n, and F. This is important because bias dependent changes in A can be influenced by c_i unless high mass transport conditions are provided. Moreover, the z charge valence in the exponent is assumed to be 1 since electron transfer reactions are assumed to occur one at a time. Conway's formalism for the temperature dependence of the symmetry coefficient[12,13] ($\beta = \beta_H + T\beta_S$) is introduced to account for the change in $\Delta S^{\#}$ and $\mathrm{E_A}$ (or $\Delta H^{\#}$):

$$j = A_0 \exp\left(\frac{-\mathrm{E}_{\mathrm{A}_0}}{RT}\right) \exp\left(\frac{[\,\beta_H + T\beta_S\,]\,F\,\eta}{RT}\right) = A_0 \exp\left(\frac{\beta_S\,F\eta}{R}\right) \exp\left(\frac{-\mathrm{E}_{\mathrm{A}_0} + \beta_H\,F\eta}{RT}\right) \quad \mathbf{(11)}$$

For a given η, a new apparent activation energy, $E_{A,app}$, and pre-exponential factor, A_{app}, may be defined as:

$$E_{A_{\mathrm{app}}}(\eta) = E_{A_0} - \beta_H\,F\eta \quad \mathbf{(12)}$$

$$\log_{10} A_{\mathrm{app}}(\eta) = log_{10} A_0 + \frac{\beta_S F\eta}{\ln(10)\,R} \quad \mathbf{(13)}$$

Both expressions are reorganized to isolate the $F\eta$ term:

$$F\eta = \frac{\mathrm{E}_{\mathrm{A}_{\mathrm{app}}} - \mathrm{E}_{\mathrm{A}_0}}{\beta_H} \quad \mathbf{(14)}$$

$$F\eta = \left[\frac{\log_{10} A_{app} - \log_{10} A_0}{\beta_S}\right] \ln(10)\,R \quad \mathbf{(15)}$$

Both expressions are equated, as the overpotential acts both on the energy of activation and the pre-exponential factor:

$$\frac{E_{A_{\mathrm{app}}} - E_{A_0}}{\beta_H} = \left[\frac{\log_{10} A_{\mathrm{app}} - \log_{10} A_0}{\beta_S}\right] \ln(10)\,R \quad \mathbf{(16)}$$

In the kinetic maps, $log_{10} A(\eta)$ is plotted on the y axis and $E_A(\eta)$ on the x axis. The previous expression may be rearranged to obtain a linear expression analogous to $\log_{10} A_{app}(\eta) = a + b\,E_{A_{\mathrm{app}}}(\eta)$:

$$\log_{10} A_{\mathrm{app}}(\eta) = \left[\log_{10} A_0 + \frac{\beta_S\,E_{A_0}}{\ln(10)\,R\,\beta_H}\right] - \frac{\beta_S}{\ln(10)\,R\,\beta_H}\,\mathrm{E}_{\mathrm{A}_{\mathrm{app}}}(\eta) \quad \mathbf{(17)}$$

This expression relates changes in the apparent pre-exponential factor for a given η with the activation energy, allowing to quantify compensation effects (Constable-Cremer relationships[14]) in electrocatalysis based on the kinetic parameters. The compensation slope predicted with this expression (b_{CE}) is:

$$b_{CE} = -\frac{\beta_S}{\ln(10)\,R\,\beta_H} \quad \mathbf{(18)}$$

Which intuitively informs on the ratio between modifying the entropic and enthalpic contributions to the free energy of activation with units of mol J^{-1}. The natural logarithm of 10 is only valid when the Arrhenius analysis and the pre-exponential factor as expressed in a base of log10. If using the natural logarithm base, the conversion factor should be taken out of the equation. Note that an entropic coefficient of $\beta_S = 0$ implies the absence of compensation slope, indicating that the applied overpotential will only reduce the activation energy (appearing as a horizontal line in the kinetic map). Moreover, this equation may be applied not only to compensation effect regions over an extended overpotential range, but given the temperature and potential-dependence of the transfer coefficients/Tafel slopes it may be applied to calculate the differential slopes in kinetic maps. Furthermore, both equilibrium

parameters, $\log_{10}A_0$ and $\mathrm{E}_{\mathrm{A}_0}$, are absent from this expression, allowing us to calculate compensation effects for any set of symmetry coefficients given their temperature-dependence. The origin intercept for the compensation effect (OI_{CE}) is:

$$OI_{CE} = \log_{10}A_0 + \frac{\beta_S\, E_{A_0}}{\ln(10)\, R\, \beta_H} = \log_{10}A_0 - b_{CE}\, \mathrm{E}_{\mathrm{A}_0} \quad \mathbf{(19)}$$

In theory, this would allow us to access the equilibrium parameters. However, this is only valid for reactions where b_{CE} does not significantly change over a substantial overpotential range.

Note that the symmetry coefficient β can only be written for a single charge transfer step and that this equation should be written in the generality with the charge transfer coefficient α, as it may account for multi-step processes and is not constrained to be defined between zero and one. Experimentally, we observe compensation effects with increasing activation energies which necessarily requires of negative α_H coefficients, questions whether the constrain between zero and one of the total β even applies to β_H and β_S. In general, without a more detailed discussion of β_H and β_S, expressions (18) and (19) should be written using the transfer coefficients. Expressions (18) and (19) become:

$$b_{CE} = -\frac{\alpha_S}{\ln(10)\, R\, \alpha_H} \quad (20)$$

$$OI_{CE} = \log_{10}A_0 + \frac{\alpha_S\, E_{A_0}}{\ln(10)\, R\, \alpha_H} = \log_{10}A_0 - b_{CE}\, \mathrm{E}_{\mathrm{A}_0} \quad (21)$$

Which are the expressions used to in the main text of this manuscript.

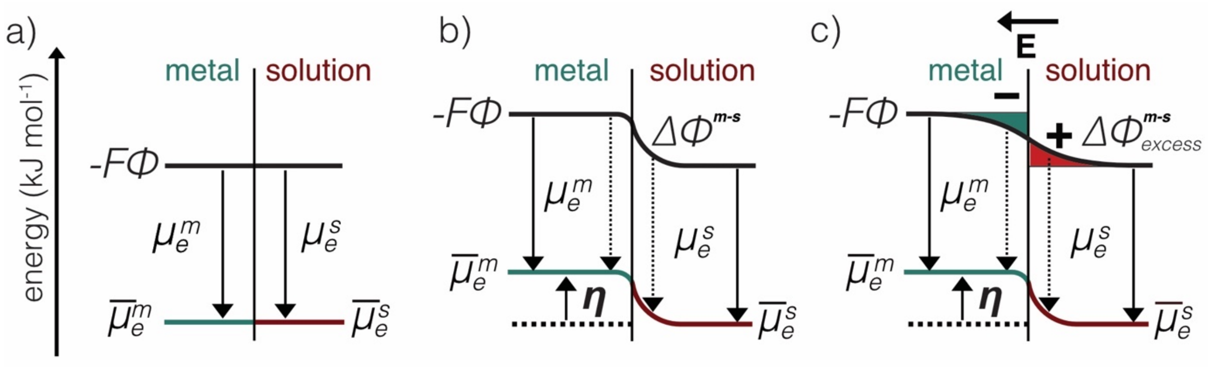

Supplementary Figure 1. The difference between a non-polarizable and polarizable metal-solution interface. (a) Electrochemical equilibrium at a metal-solution interface where the equilibrium potential matches the potential of zero free charge. The electrochemical potentials of the electrons in the metal, $\bar{\mu}_e^m$, and in the solution[15], $\bar{\mu}_e^s$, are aligned and the (local) chemical potentials of the electrons in the two phases, μ_e^m and μ_e^s, are identical. (b) At an applied overpotential, η, the local chemical potentials of the electron stay constant (the solid and dashed arrows have the same lengths in the bulk and at the interface). This constitutes an ideally non-polarizable interface. Under this condition, the applied overpotential is translated into an electrostatic potential drop, $\Delta\bar{\mu}_e^{m-s} = -F\Delta\Phi^{m-s}$. (c) At an applied overpotential, the local chemical potentials might change. The electrostatic potential difference that builds up at the interface, $\Delta\Phi_{excess}^{m-s}$, is related to an electric field across a double layer composed of water dipoles and ions. To translate this picture to the bipolar membrane (BPM), one needs to distinguish the oxide catalyzed case from the one of the pristine BPM. In the former case, the active sites are the ones on the non-conductive metal oxides, that can be charged depending on the local pH and the point of zero charge. However, the applied overpotential does not additionally polarize the non-conductive oxides. In contrast, for the pristine BPM, there exists already an electric field at electrochemical equilibrium. With applied overpotential, this equilibrium field is either unaltered, assuming a constant protonation or hydroxylation state of the space charge region, or the latter is polarized and changes its excess charge dependent on the direction of the overpotential.

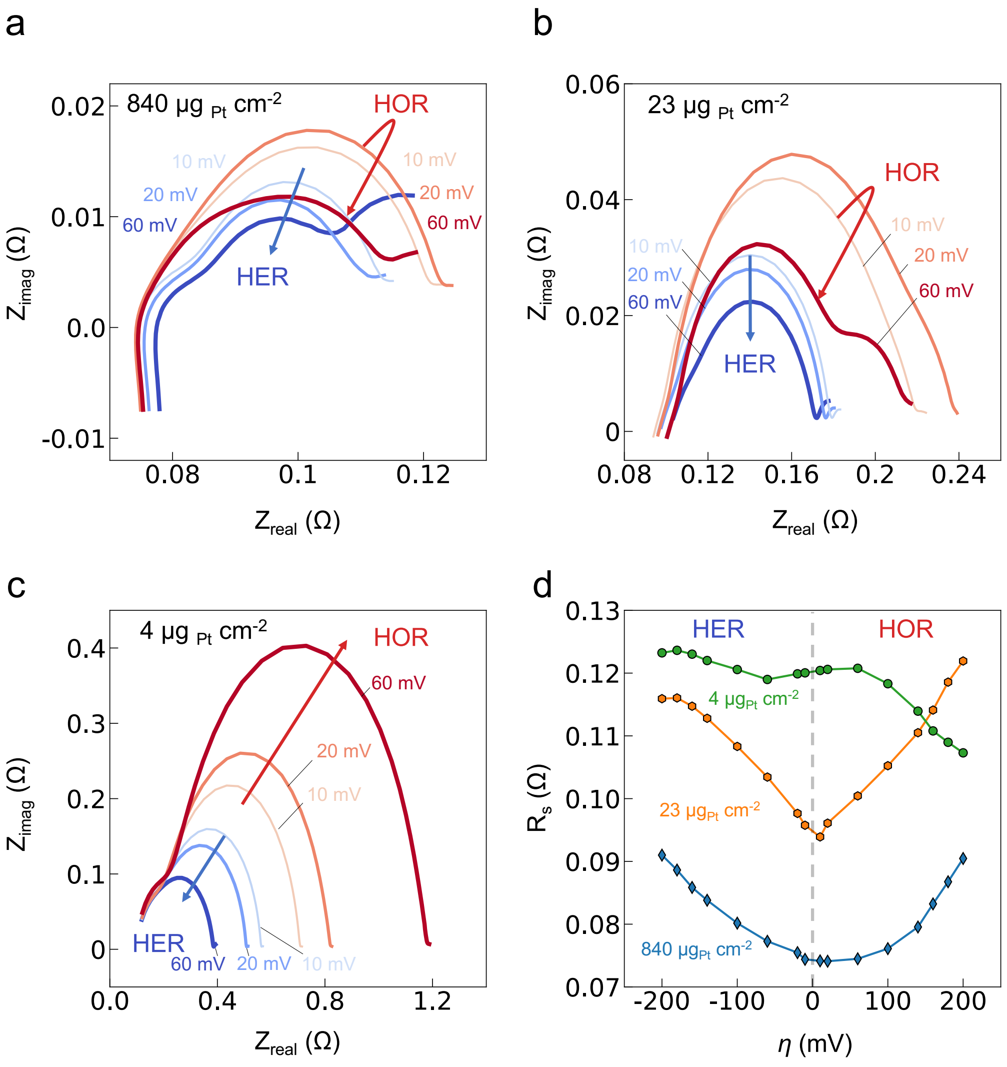

Supplementary Figure 2. Electrochemical impedance spectroscopy for different working electrode loadings in the HER/HOR H_2-pump cell. a, High loading (840 μg_{Pt} cm^{-2}) working electrode. The impedance shows inductive features (negative Z_{imag}) as the loading matches that of the counter electrode. In this case, the current potential response is simultaneously influenced by the working and counter electrodes and by the proton transport through the membrane. **b,** Medium loading (23 μg_{Pt} cm^{-2}) chosen for the kinetic studies in this work. Both the HER and HOR overpotentials behave similarly. **c,** Low loading (4 μg_{Pt} cm^{-2}) working electrode showing a clear asymmetric behavior between the HER and HOR. At lower loadings, lateral proton transport across the ionomer to sparse active sites might be impacted by different water transport conditions between HER and HOR. **d,** Extracted series resistance (R_s) from high frequency Z_{real}. The similar and low values obtained for every loading highlight the high mass transport of the H_2-pump. Frequency range of 1-10^6 Hz at 55 ˚C.

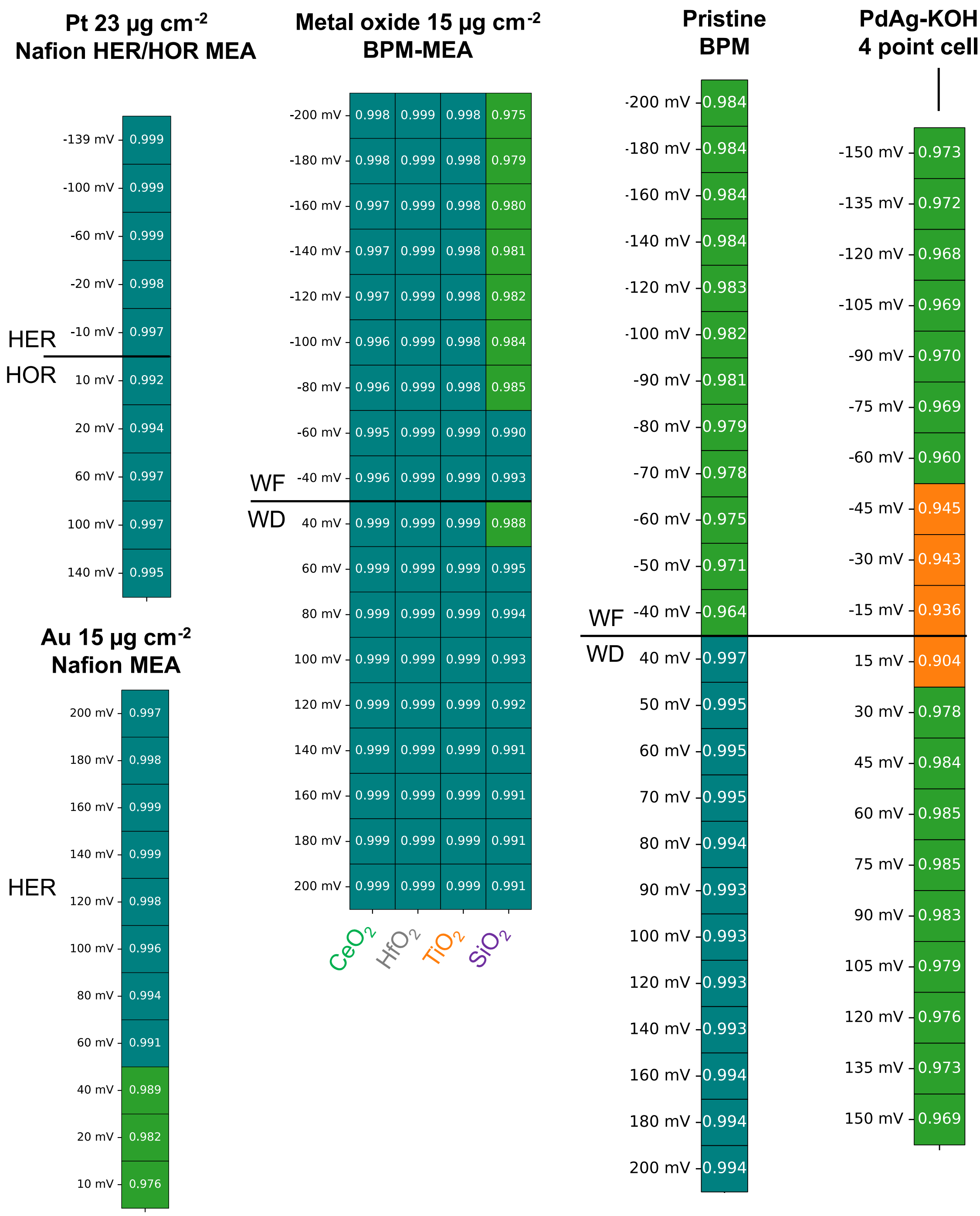

Supplementary Figure 3. Heatmaps for the R^2 coefficients for the linear Arrhenius regressions. The goodness of a linear regression is generally estimated by the R^2 coefficient. The precision of the linear regression, that is, the deviation of the points from the trendline is estimated by the standard error of the regression (S), plotted in each kinetic map as the error bars of $\log_{10}A$ and E_A. Except for the PdAg foil, the R^2 coefficients are above 0.95, showing the advantage and robustness of the membrane-electrode assembly (MEA) setups for temperature-dependent studies. For all experiments, 5 temperatures were used for the Arrhenius fit. The color code is used to emphasize different R^2 values (teal ≥ 0.99, light-green ≥ 0.95, orange ≥ 0.9).

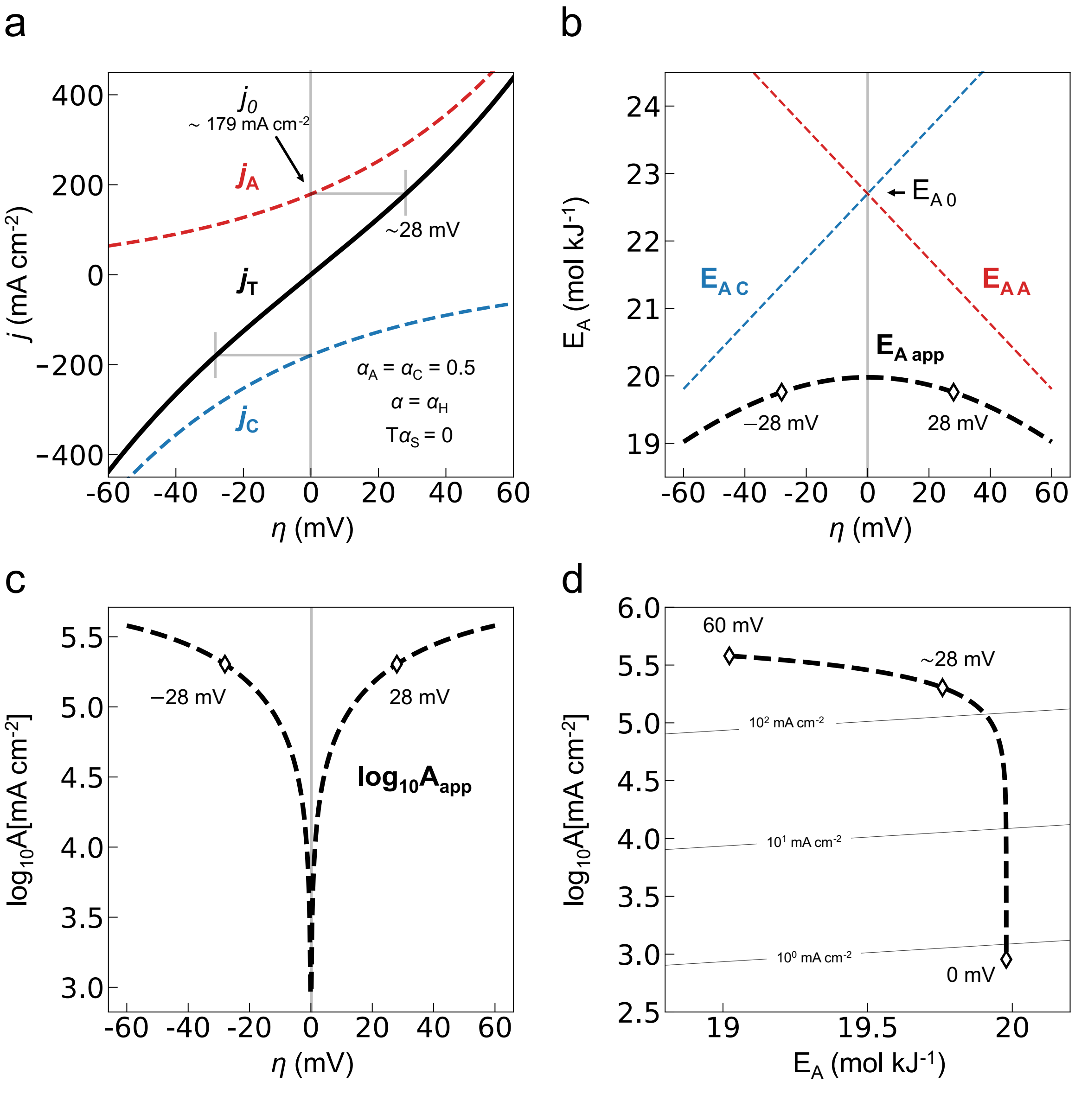

Supplementary Figure 4. Simulated kinetics of a system with bias- and temperature-independent transfer coefficients that are the same for the anodic and cathodic reaction ($\boldsymbol{\alpha(\eta, T) = \alpha_H}$). a, Total and measured current density (j_T) and partial anodic (j_A) and cathodic (j_C) current densities for a simulated system with $\alpha_H = 0.5$. At an overpotential of ± 28 mV, j_T reaches the value of the exchange current density where the influence of the backward reaction still has a significant influence on the total current density. **b,** Apparent $E_{A,app}$), cathodic ($E_{A,C}$), and anodic activation energy ($E_{A,A}$). **c,** Apparent pre-exponential factor ($\log A_{app}$) calculated from j_T. The apparent $\log A_{app}$ increases for low overpotentials, as there is a simultaneous enhancement (suppression) of the forward (backward) reaction by a decreasing

(increasing) activation energy. **d,** Kinetic map for reversible catalyst with apparent constant activation energy and rising pre-exponential factor.

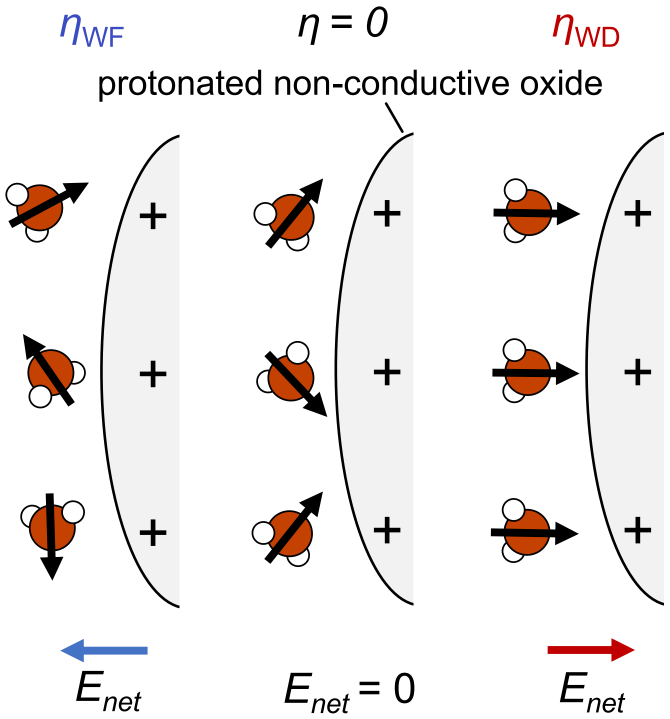

Supplementary Figure 5. Interaction between the local excess charge and net electric fields across the junction. The electric field inside the BPM might either polarize the water molecules additionally into the direction given by the excess charge on the non-conductive oxide (red arrow), as shown here for the water dissociation direction, or the net electric field can counteract the local polarization (blue arrow), as shown for the water formation direction.

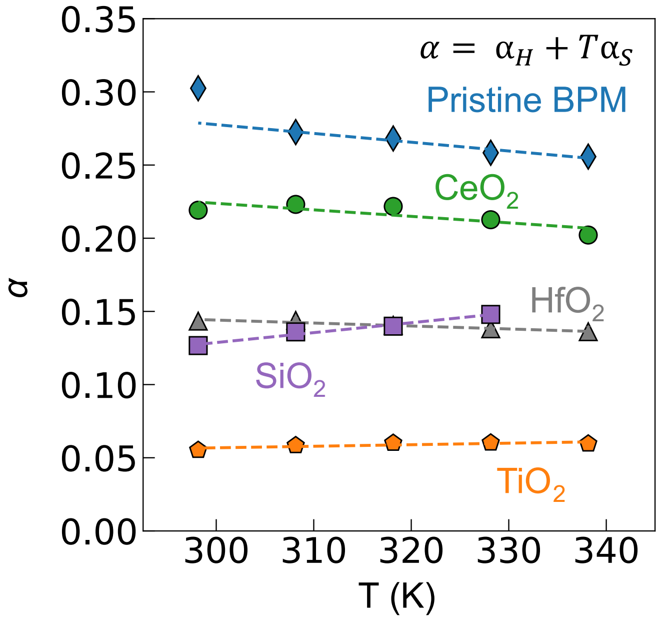

Supplementary Figure 6. Temperature-dependent charge transfer coefficients for the water formation reaction ($H_2O \rightarrow OH^- + H^+$) for bipolar membranes containing different metal oxides. With decreasing point of zero charge ($CeO_2 \approx 8.1 > HfO_2 \approx 7.4 > TiO_2 \approx 6.8 > SiO_2 \approx 2.8$) the transfer coefficients decrease together in magnitude and in dependence to the temperature until it becomes small and temperature independent for TiO_2.

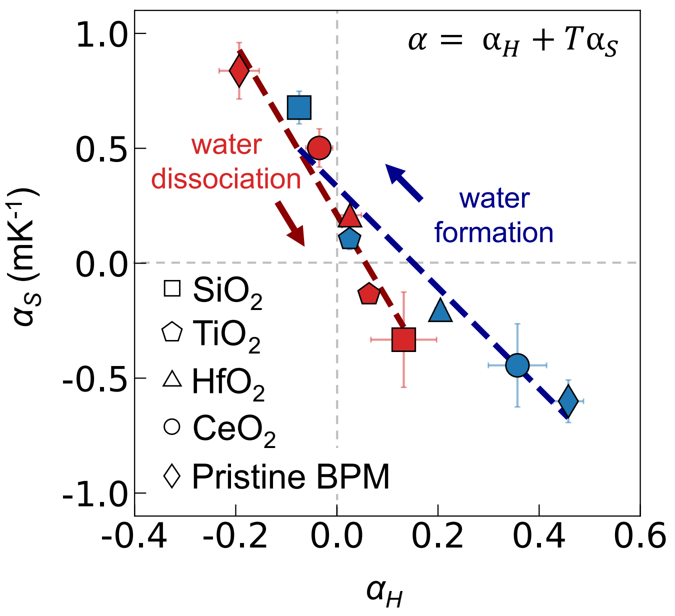

Supplementary Figure 7. Enthalpic (α_H) and entropic (α_S) components of the charge transfer coefficient (α) for bipolar membrane junctions containing different metal oxides. Both for water dissociation and water formation a compensation is found where the coefficients gradually change with decreasing point of zero charge ($CeO_2 \approx 8.1 > HfO_2 \approx 7.4 > TiO_2 \approx 6.8 > SiO_2 \approx 2.8$) of the metal-oxide nanoparticles employed. The more acidic the nanoparticles, the more reversible the BPM junction becomes. However, we find that this reversibility comes by decreasing α_S and increasing α_H for WD, while for WF it is the opposite, increasing α_S (making it less negative) and increasing α_H, suggesting that the bias affects the free energy barrier for both of these reactions differently.

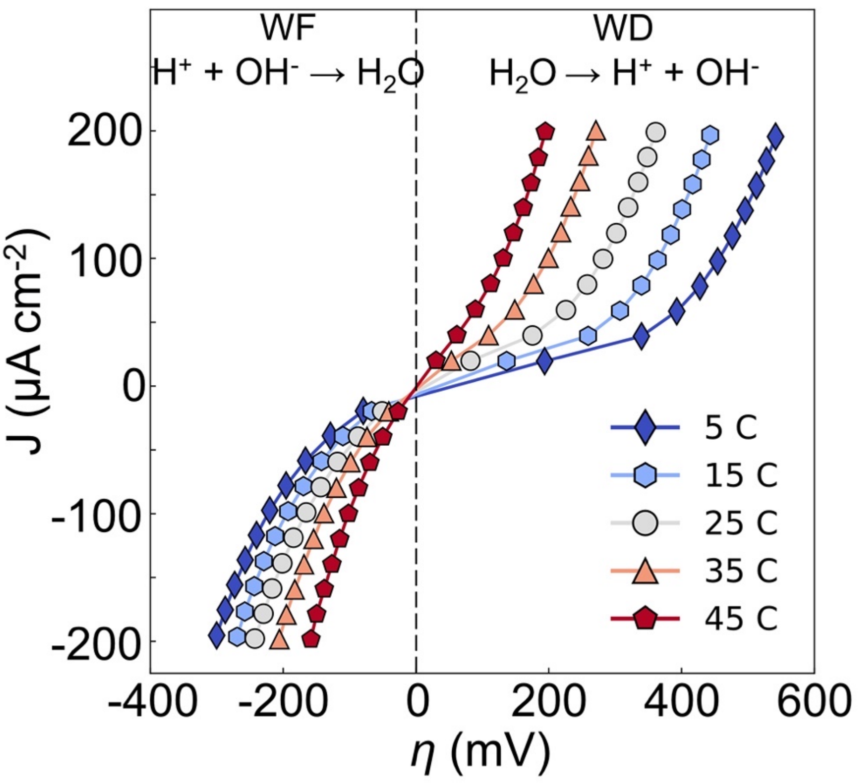

Supplementary Figure 8. Water dissociation and formation polarization curves for different temperatures of the PdAg-KOH junction. For a schematic of the cell design, see Fig. 4c in the main.

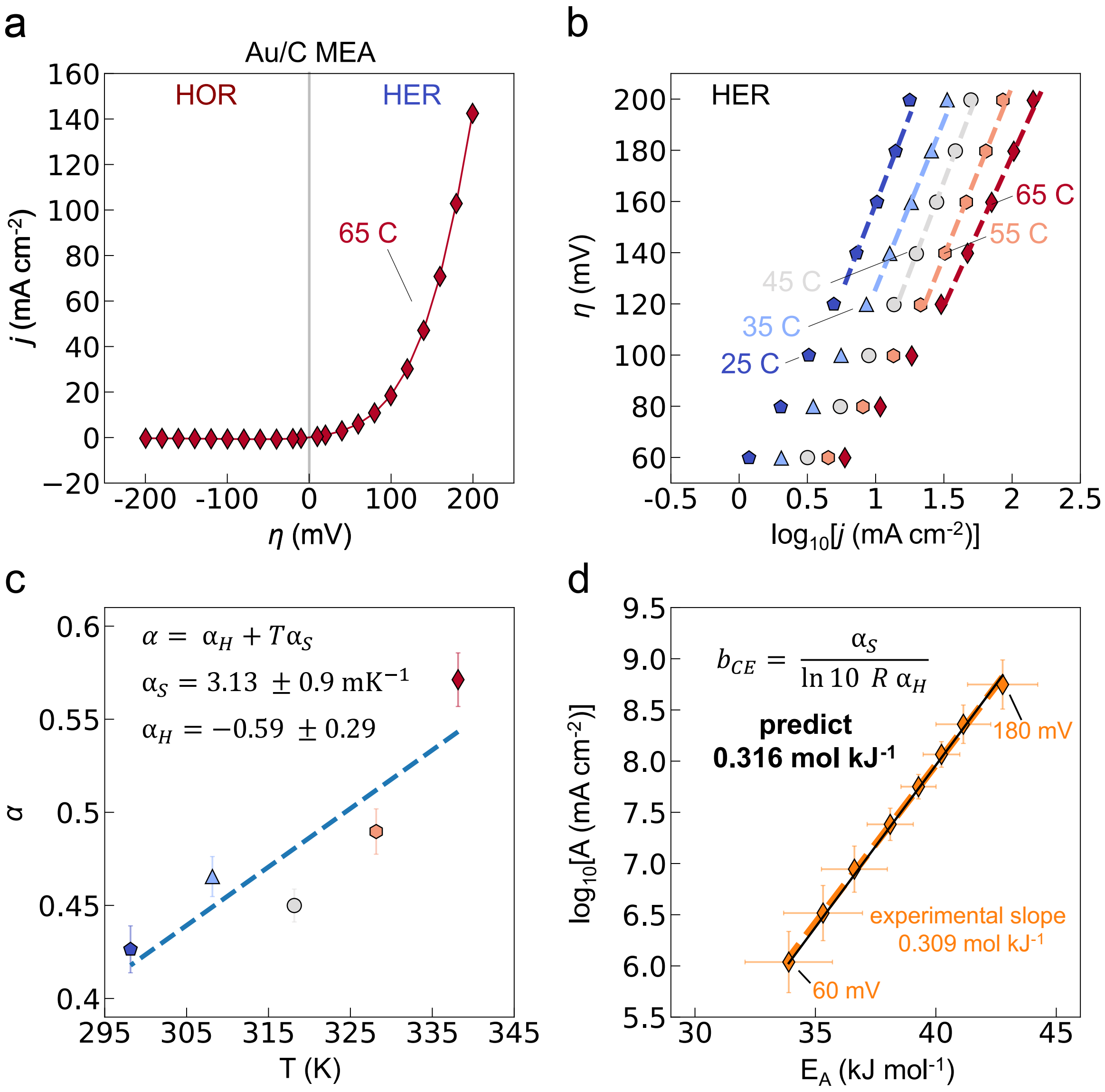

Supplementary Figure 9. Irreversible hydrogen evolution reaction kinetics on Au nanoparticles (15 µg cm^{-2}) in an acidic Nafion MEA and estimation of the compensation. **a,** Polarization curve (polarity of the potential and currents are flipped) at 65˚C showing the strong irreversibility of Au towards the HER/HOR pair. The first step for the HOR reaction is the dissociative adsorption step (H_2 + 2M* → 2M-H), which does not occur on Au. **b,** η vs $\log_{10} j$ plot where the Tafel slope (b_T) can be calculated for different temperatures for the HER. **c,** Temperature-dependent charge transfer coefficients (α) and their fit to Conway's formalism to obtain their enthalpic (α_H) and entropic (α_S) components for the HER. **d,** Kinetic map showing the Arrhenius compensation for HER on Au and the prediction according to the charge transfer coefficients (**equation (5)**) in the main. HER data on Au was previously published[16].

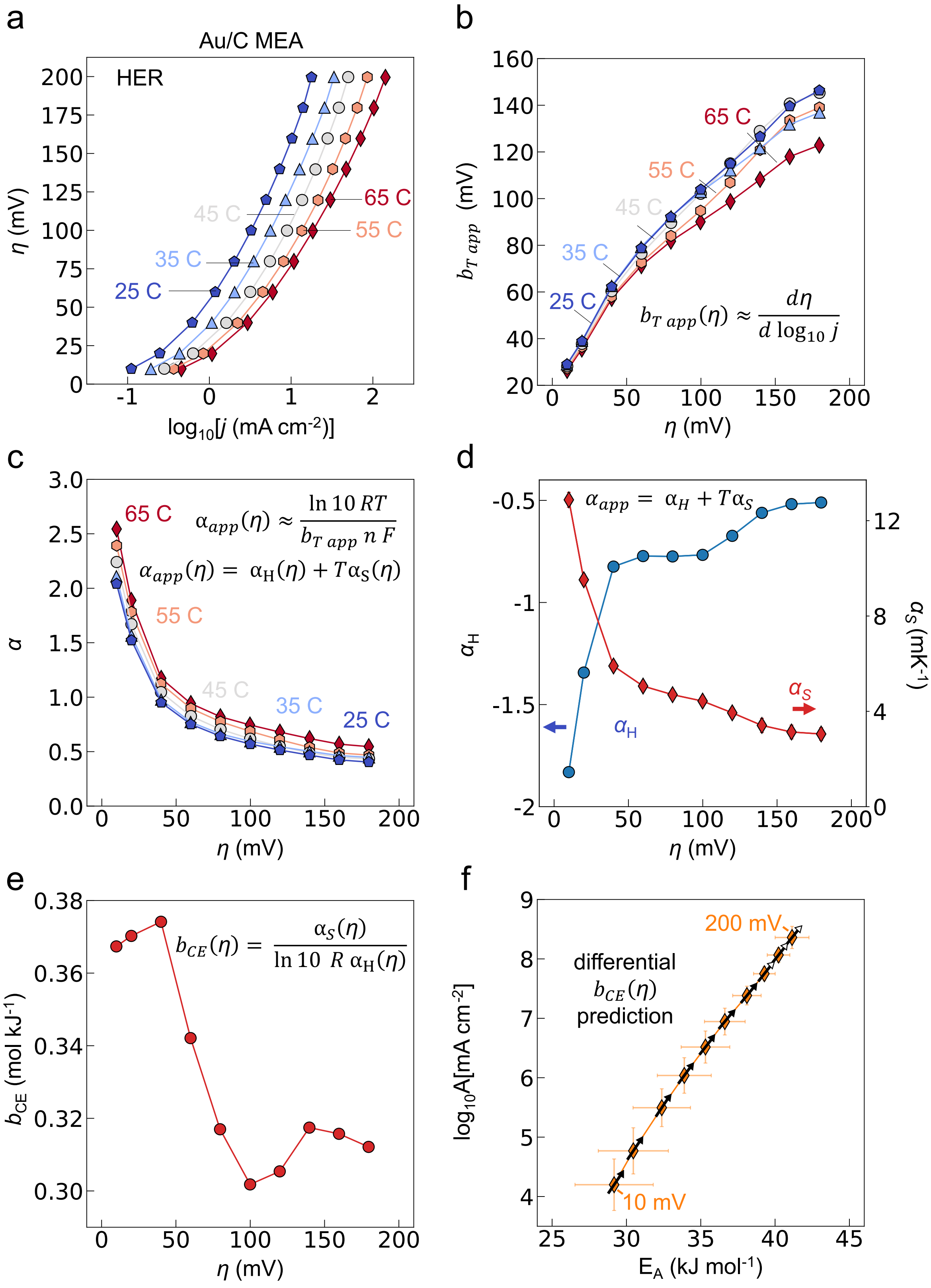

Supplementary Figure 10. Irreversible hydrogen evolution reaction kinetics on Au nanoparticles (15 μg cm^{-2}) in an acidic Nafion MEA and estimation of the compensation. a, For each temperature, the central gradient of η vs $\log_{10}j$ yields an apparent Tafel slope ($b_{T\ app}$). **b,** Apparent transfer coefficient (α_{app}) for each temperature, allowing us to estimate

and apparent enthalpic and entropic transfer contributions *via* Conway's formalism. Note, the term 'apparent', as the charge transfer coefficient is just extracted from the gradient and not obtained by fitting the data to a kinetic expression. While the 'true' transfer coefficient may be potential dependent, we note that the apparent one comes from the gradual change in the logarithmic current scale. **c-d,** The temperature-dependence of α_{app} can be used to calculate an enthalpic and entropic contribution at each potential. Here, α_H is negative, indicating that the potential modulation increases the activation energy. **e,** Differential compensation slope (b_{CE}) predicted for each potential using α_S and α_H with **equation (5)** of the main text. **f,** Differential slope on top of the kinetic map points (black arrows), showing that this slope accurately describes the direction followed when increasing the potential in the kinetics. Despite using apparent Tafel and transfer coefficients obtained by a numerical gradient, the temperature-dependence of these parameters carries information regarding the compensatory kinetics as these may be used to accurately describe the differential slopes that connect each point in the kinetic map. HER data on Au was previously published by us[16].

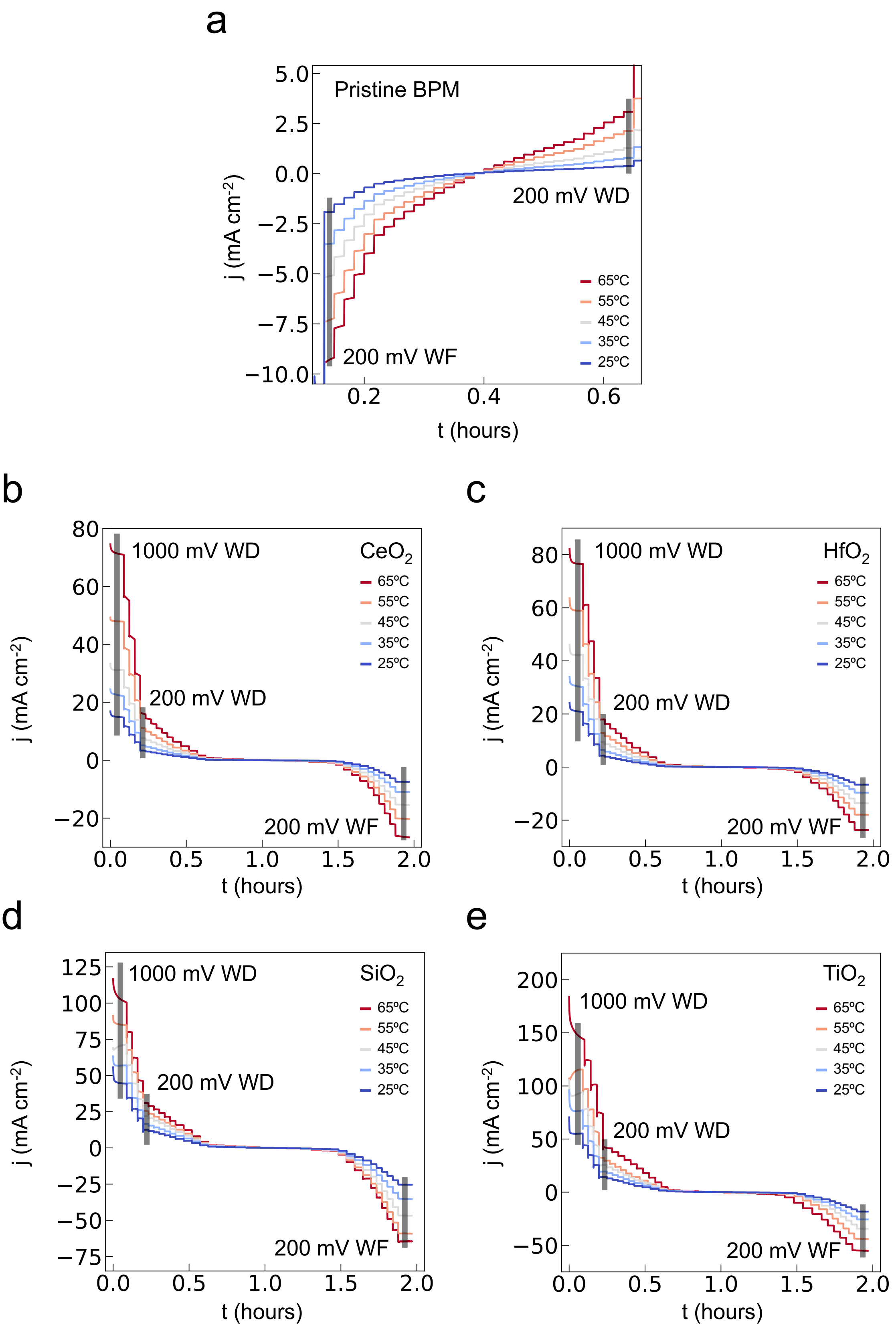

Supplementary Figure 11. Temperature dependent chronoamperometry. **A**, Pristine BPM. The results are zoomed in around the relevant ±200 mV WD/WF range to inspect the stability of the data in the relevant range, where the kinetic analysis is performed. The direction of

measurement from WF to WD does not affect the stability of the results. The measurement protocol begins at 1000 mV WF and ends in 1000 mV WD, lasting a total of 45 minutes (0.75 hours). **b-e**, Chronoamperometry for the BPMs containing different metal oxides in the junction. The protocol beings at 1000 mV WD. The initial WD steps are generally marked by a transient behavior and are not employed in the kinetic analysis, restricting the interpretation of results in the range of ±200 mV WD/WF to stay close to electrochemical equilibrium, avoiding the influence of the change of the reactant concentration or product water concentration on the electrochemical rate. Moreover, the steps in this range show a reproducible stable behavior over 2 hours at different temperatures, suggesting a minimal influence overall of catalyst changes or degradation across temperatures. This is especially relevant for metal oxides such as SiO_2, where a constant influx particularly of OH^- during the forward-bias WF may dissolve part of the catalyst potentially altering kinetics. For this reason, the temperature and potential range was restricted in these studies, limiting the measurement to -200 mV WF and reaching temperatures as high as 65ºC. Nevertheless, the operation of BPM fuel cells and electrolyzers at high current densities and temperatures may be impacted by such effects, requiring further stability test for each metal oxide at elevated temperatures.

Supplementary References

1. Tang, B. Y., Bisbey, R. P., Lodaya, K. M., Toh, W. L. & Surendranath, Y. Reaction environment impacts charge transfer but not chemical reaction steps in hydrogen evolution catalysis. *Nat. Catal.* **6**, 339–350 (2023).
2. Durst, J., Simon, C., Hasché, F. & Gasteiger, H. A. Hydrogen Oxidation and Evolution Reaction Kinetics on Carbon Supported Pt, Ir, Rh, and Pd Electrocatalysts in Acidic Media. *J. Electrochem. Soc.* **162**, F190 (2014).
3. Eliaz, N. & Gileadi, E. *Physical Electrochemistry: Fundamentals, Techniques, and Applications*. (Wiley).
4. Guidelli, R. *et al.* Defining the transfer coefficient in electrochemistry: An assessment (IUPAC Technical Report). *Pure Appl. Chem.* **86**, 245–258 (2014).
5. Agar, J. N. The interpretation of overpotential measurements. *Discuss. Faraday Soc.* **1**, 81–86 (1947).
6. Conway, B. E. & Wilkinson, D. F. Entropic and enthalpic components of the symmetry factor for electrochemical proton transfer from various proton donors over a wide temperature range. *J. Electroanal. Chem. Interfacial Electrochem.* **214**, 633–653 (1986).
7. Conway, B. E., Tessier, D. F. & Wilkinson, D. P. Experimental evidence for the potential-dependence of entropy of activation in electrochemical reactions in relations to the temperature-dependence of tafel slopes. *J. Electroanal. Chem. Interfacial Electrochem.* **199**, 249–269 (1986).
8. Conway, B. E., MacKinnon, D. J. & Tilak, B. V. Significance of electrochemical Brønsted factors. Kinetic studies over a wide range of temperatures. *Trans Faraday Soc* **66**, 1203–1226 (1970).
9. Conway, B. E., Phillips, Y. & Qian, S. Y. Surface electrochemistry and kinetics of anodic bromine formation at platinum. *J. Chem. Soc. Faraday Trans.* **91**, 283–293 (1995).
10. Teschner, D. *et al.* In situ surface coverage analysis of RuO2-catalysed HCl oxidation reveals the entropic origin of compensation in heterogeneous catalysis. *Nat. Chem.* **4**, 739–745 (2012).
11. Gileadi, E. & Kirowa-Eisner, E. Some observations concerning the Tafel equation and its relevance to charge transfer in corrosion. *Corros. Sci.* **47**, 3068–3085 (2005).
12. Conway, B. E., Tessier, D. F. & Wilkinson, D. P. Temperature Dependence of the Tafel Slope and Electrochemical Barrier Symmetry Factor, β, in Electrode Kinetics. *J. Electrochem. Soc.* **136**, 2486 (1989).
13. Conway, B. E. & Wilkinson, D. F. Entropic and enthalpic components of the symmetry factor for electrochemical proton transfer from various proton donors over a wide temperature range. *J. Electroanal. Chem. Interfacial Electrochem.* **214**, 633–653 (1986).
14. Liu, L. & Guo, Q.-X. Isokinetic Relationship, Isoequilibrium Relationship, and Enthalpy−Entropy Compensation. *Chem. Rev.* **101**, 673–696 (2001).
15. Boettcher, S. W. *et al.* Potentially Confusing: Potentials in Electrochemistry. *ACS Energy Lett.* **6**, 261–266 (2021).
16. Gisbert-González, J. M. *et al.* Bias Dependence of the Transition State of the Hydrogen Evolution Reaction. *J. Am. Chem. Soc.* https://doi.org/10.1021/jacs.4c18638 (2025) doi:10.1021/jacs.4c18638.
